# Entropy and the Tolman Parameter in Nucleation Theory

**DOI:** 10.3390/e21070670

**Published:** 2019-07-09

**Authors:** Jürn W. P. Schmelzer, Alexander S. Abyzov, Vladimir G. Baidakov

**Affiliations:** 1 Institute of Physics, University of Rostock, Albert-Einstein-Strasse 23-25, 18059 Rostock, Germany; 2 National Science Center Kharkov Institute of Physics and Technology, 61108 Kharkov, Ukraine; 3 Institute of Thermal Physics, Ural Branch of the Russian Academy of Sciences, Amundsen Street 107a, 620016 Yekaterinburg, Russia

**Keywords:** crystallization, segregation, condensation, boiling, nucleation, curvature dependence of the surface tension, 64.60.Bd General theory of phase transitions, 64.60.Q Nucleation, 68.35.Md Surface thermodynamics, surface energies in surfaces, and interfaces, 82.60.Nh Thermodynamics of nucleation in physical chemistry and chemical physics

## Abstract

Thermodynamic aspects of the theory of nucleation are commonly considered employing Gibbs’ theory of interfacial phenomena and its generalizations. Utilizing Gibbs’ theory, the bulk parameters of the critical clusters governing nucleation can be uniquely determined for any metastable state of the ambient phase. As a rule, they turn out in such treatment to be widely similar to the properties of the newly-evolving macroscopic phases. Consequently, the major tool to resolve problems concerning the accuracy of theoretical predictions of nucleation rates and related characteristics of the nucleation process consists of an approach with the introduction of the size or curvature dependence of the surface tension. In the description of crystallization, this quantity has been expressed frequently via changes of entropy (or enthalpy) in crystallization, i.e., via the latent heat of melting or crystallization. Such a correlation between the capillarity phenomena and entropy changes was originally advanced by Stefan considering condensation and evaporation. It is known in the application to crystal nucleation as the Skapski–Turnbull relation. This relation, by mentioned reasons more correctly denoted as the Stefan–Skapski–Turnbull rule, was expanded by some of us quite recently to the description of the surface tension not only for phase equilibrium at planar interfaces, but to the description of the surface tension of critical clusters and its size or curvature dependence. This dependence is frequently expressed by a relation derived by Tolman. As shown by us, the Tolman equation can be employed for the description of the surface tension not only for condensation and boiling in one-component systems caused by variations of pressure (analyzed by Gibbs and Tolman), but generally also for phase formation caused by variations of temperature. Beyond this particular application, it can be utilized for multi-component systems provided the composition of the ambient phase is kept constant and variations of either pressure or temperature do not result in variations of the composition of the critical clusters. The latter requirement is one of the basic assumptions of classical nucleation theory. For this reason, it is only natural to use it also for the specification of the size dependence of the surface tension. Our method, relying on the Stefan–Skapski–Turnbull rule, allows one to determine the dependence of the surface tension on pressure and temperature or, alternatively, the Tolman parameter in his equation. In the present paper, we expand this approach and compare it with alternative methods of the description of the size-dependence of the surface tension and, as far as it is possible to use the Tolman equation, of the specification of the Tolman parameter. Applying these ideas to condensation and boiling, we derive a relation for the curvature dependence of the surface tension covering the whole range of metastable initial states from the binodal curve to the spinodal curve.

## 1. Introduction

The classical theory of nucleation and growth processes (CNT) is in different forms till now one of the major tools in the interpretation of experimental data on the kinetics of first-order phase transitions. It is based in its thermodynamic ingredients on the thermodynamic description of interfacial phenomena as developed by Josiah W. Gibbs [1]. Gibbs’ method is utilized in CNT for two purposes, the determination of the thermodynamic driving force of phase formation and the account of interfacial terms in the specification of the work of critical cluster formation.

Utilizing Gibbs’ classical treatment in the description of thermodynamically-heterogeneous systems (for alternative approaches, see [2,3,4,5,6,7]), different methods can be employed for the computation of the thermodynamic driving force of the transformation. In terms of Gibbs’ theory, the thermodynamic driving force of nucleation can be determined with high accuracy provided sufficient information about the systems under consideration is available. Even in cases when such comprehensive information is not at one’s disposal and the driving force has to be estimated by approximative methods, it is supposed that the approximations are widely correct. For this reason, the major tool to resolve problems concerning the accuracy of theoretical predictions of nucleation rates and related characteristics of the nucleation process consists of the introduction of the size or curvature dependence of the surface tension.

Such an approach was originally suggested also already by Gibbs [1] and then followed and extended by many others. In such treatment, the dependence of the surface tension, σ, on the radius, *R*, of the critical clusters can be determined at certain conditions specified below by the differential equation [8,9,10,11,12,13]:

(1) $$\frac{d\sigma }{\sigma }=-\frac{2\delta }{1+\frac{2\delta }{R}}d\left(\frac{1}{R}\right)\;.$$

For the case considered first by Gibbs [1] and elaborated in detail by Tolman [12] (isothermal condensation and boiling in one-component fluids caused by variations of pressure), the function δ(R) is given by:

(2) $$\delta \left(R\right)=\left(R_{e}-R\right)\left[1+\frac{\left(R_{e}-R\right)}{R}+\frac{1}{3}{\left(\frac{\left(R_{e}-R\right)}{R}\right)}^{2}\right]\;.$$

These relations are a direct consequence of Gibbs’ equilibrium conditions (equality of the temperature and chemical potentials of the different components, the Young–Laplace equation) and Gibbs’ adsorption equation. Some details of their derivation will be given below.

In the above equations, *R* is the radius of the critical cluster (assumed to be of a spherical shape, where the surface tension [1] is chosen as the dividing surface) and σ(R) is the surface tension for a cluster of critical size for this particular dividing surface. The radius of the equimolecular dividing surface [1] is denoted by $R_{e}$. It is also located inside the inhomogeneous region between the coexisting phases. For this reason, the absolute value of $\delta_{\infty }=\delta \left(R\to \infty \right)$ has to be less than the width of the inhomogeneous region in between them. The solution of Equation (1) depends on the function δ(R). Its value, $\delta_{\infty }=\delta \left(R\to \infty \right)$, ie., in the limit of large critical cluster sizes, it is commonly denoted as the Tolman parameter.

Based on the above given general relations, a set of approximate expressions can be derived for the curvature dependence of the surface tension employing different assumptions concerning the function δ=δ(R) [8,9,10,11,12,13]. One of the equations widely utilized in the description of such size or curvature dependence of the surface tension is a relation suggested by Tolman [12],

(3) $$\sigma \left(R\right)=\frac{\sigma_{\infty }}{1+\frac{2\delta }{R}}\;,\;\sigma_{\infty }=\sigma_{\infty }\left(T_{eq},p_{eq}\right)\;,\;\delta =\delta_{\infty }\left(T_{eq},p_{eq}\right)\;.$$

It results from Equation (1) when the assumption $\delta =\delta_{\infty }=$ constant are introduced. In the above relation, $\sigma_{\infty }$ is the value of the surface tension for an equilibrium coexistence of both phases at a planar interface with values of pressure and temperature specified by the subscript (eq). In the approximation described by Equation (3), the Tolman parameter, $\delta_{\infty }$, is, consequently, the main parameter determining the curvature dependence of the surface tension. For this reason, a large number of studies have been devoted to its determination [14,15,16,17,18,19,20,21,22]. It is a quantity that has to be defined for the respective states of the equilibrium coexistence of both phases at a planar interface. In application to condensation and boiling, equilibrium is realized for states along the binodal curves [23] characterized by a temperature, $T_{b}$, and a pressure, $p_{b}$, i.e., $\sigma_{\infty }=\sigma_{\infty }\left(T_{b},p_{b}\right)$. The Tolman parameter is, in general, also a function of $T_{b}$ and $p_{b}$, i.e., $\delta_{\infty }=\delta \left(R\to \infty \right)=\delta_{\infty }\left(T_{b},p_{b}\right)$. For crystallization of liquids, we have similarly $\delta_{\infty }=\delta \left(R\to \infty \right)=\delta_{\infty }\left(T_{m},p_{m}\right)$, where $T_{m}$ and $p_{m}$ are the melting (liquidus) temperature and melting pressure for some selected equilibrium state.

The first of the main aims of the present paper consists of the further development of a thermodynamic method of the determination of the Tolman parameter relying on entropy concepts. The method results in equations requiring only directly-measurable data on the state parameters of the coexisting phases and its comparison with alternative methods of the specification of its value. For such purposes, we will employ the method of the derivation of expressions for the surface tension, σ(T,p), for the values of temperature, *T*, and pressure, *p*, different from the respective values along the melting or binodal curves derived by some of us in [24,25,26] in the application to crystallization. At such conditions, thermodynamic equilibrium can be also realized, but only for critical clusters (e.g., drops and bubbles or crystallites) of finite sizes provided that Gibbs’ equilibrium conditions can be fulfilled. In this analysis, we will go beyond Tolman’s approach considering not only phase formation caused by the variation of pressure, but also similar processes caused by the variation of temperature. In addition, we consider multi-component systems specifying the conditions at which the curvature dependence of the surface tension can be described by the differential equation, Equation (1), and consequently, the Tolman equation can be employed as an approximative description of this dependence.

As is evident from the above-given relations, the Tolman equation is an approximation valid, as a rule, for small deviations from thermodynamic equilibrium, only. For this reason, it may not be sufficiently accurate in order to describe nucleation proceeding with measurable rates only at sufficiently large supersaturations (see also [15]). An overview of the advantages and limitations of the Tolman equation in its application to condensation and boiling can be found in [12,13,16,17,18,27,28,29]. As demonstrated there, an appropriate description of the curvature dependence of the surface tension in the analysis of the nucleation of droplets and bubbles requires an expansion of the Tolman equation. Such a generalization can be written in the form (see [15,28,29] and the references cited therein):

(4) $$\sigma \left(R\right)=\frac{\sigma_{\infty }}{1+\frac{2\delta_{\infty }}{R}+{\left(\frac{l_{\infty }}{R}\right)}^{2}+\ldots }\;.$$

The second of the main aims of the present paper consists of the development of a thermodynamic method of determination of this second parameter, $l_{\infty }$, employing, similarly to the specification of $\delta_{\infty }$, only directly-measurable data on the bulk state parameters of the coexisting phases and its comparison with alternative methods of the specification of its value. In [15], such a procedure was performed in the application to crystal nucleation, and here, we extend the method to condensation and boiling, advancing a new equation for the determination of the surface tension valid in the whole range of metastable initial states between the binodal and spinodal curves of the ambient fluid.

Generalizations of the Tolman equation, Equation (3), like Equation (4), can be rewritten in the alternative but equivalent way as:

(5) $$\sigma \left(R\right)=\frac{\sigma_{\infty }}{1+\frac{2\delta }{R}}\;,\;\delta =\delta_{\infty }\left(1+\frac{l_{\infty }^{2}}{2\delta_{\infty }R}+\ldots \right)\;,$$

resulting in a relation formally identical to Equation (3), but with a quite different meaning of the parameter, δ. In such a more general approach, the parameter δ in Equation (3) has to be treated as a function of the critical cluster size. It follows as a consequence that by utilizing the Tolman equation, Equation (3), for the interpretation of experimental data employing δ as a fit parameter and not setting it equal to $\delta_{\infty }$, actually not the Tolman equation, but its generalization given by Equations (4) or (5) is employed. This is the reason for the success of the application of the Tolman equation in the interpretation of data on the nucleation of drops and bubbles. In detail, this topic was addressed in [15,29]. It is also the origin of the fact that experimental data on crystal nucleation can be explained by introducing the Tolman equation into the description [2,30,31,32,33,34].

In the present paper, we extend the analysis of the applicability of the Tolman equation to the description of the curvature dependence of the surface tension in nucleation. First, we review some basic results on the application of the Tolman equation to crystallization in multi-component systems as discussed by us in [15] and extend them. In particular, we compare our results with the consequences from different approaches by molecular dynamics simulations and, in particular, the methods proposed in [20,21,22]. In our approach, essentially the Stefan–Skapski–Turnbull [24] rule is utilized for the specification of the Tolman parameter. It connects capillarity phenomena and entropy changes in the considered phase transition. Having in mind the outstanding rule of the entropy concepts in different related problems [35,36,37,38,39,40,41], such an approach can be considered already in advance as highly prospective. An extension of our method to ice-crystal nucleation in water will be presented in an accompanying paper [42]. The Stefan–Skapski–Turnbull rule was derived first by Stefan [43] in application to condensation and boiling. For this reason, we will also check here what are the results of the application of our generalization of this rule to nucleation in condensation and boiling. Here, we will employ as a model condensation and boiling in van der Waals gases. The results are compared and shown to be in agreement with density functional methods originally developed by van der Waals [44,45].

The paper is structured as follows. In Section 2, based on the theory of interfacial phenomena developed by Gibbs, different approaches in the determination of the thermodynamic driving force of a phase transformation are briefly reviewed and methods of the derivation of the temperature and pressure dependence of the surface tension both for one- and multi-component systems are outlined, advancing the analysis performed in [2,24,25,26]. The conditions are specified for which the curvature dependence of the surface tension can be described by the differential equation, Equation (1), and the resulting approximate solution, Equation (3), proposed by Tolman. In Section 3, specific features in the application of these methods to crystallization are discussed, and a comparison of the results with experimental data and computer simulations is performed. In Section 4, the method of the determination of the parameters $\delta_{\infty }$ and $l_{\infty }$, respectively, similar quantities, is outlined in application to condensation and boiling, advancing a more appropriate for this case relation for the curvature dependence of the surface tension as compared to Equations (4) and (5). A summary of the results and their discussion (Section 5) completes the paper.

## 2. Thermodynamic Aspects of Nucleation: Some General Considerations

### 2.1. Thermodynamic Driving Force in Nucleation

The properties of the critical clusters in nucleation theory are determined by the necessary thermodynamic equilibrium conditions. Employing Gibbs’ classical theory [1,2,46,47], for a spherical critical cluster (with a radius, *R*, referring to the surface tension), these conditions read:

(6) $$T_{\alpha }=T_{\beta }=T\;,\;\mu_{i\alpha }=\mu_{i\beta }=\mu_{i}\;,\;p_{\alpha }-p_{\beta }=\frac{2\sigma }{R}\;.$$

Here, *T* is the temperature, *p* the pressure, $\mu_{i}$ the chemical potentials of the different components, i=1,2,…,k, where *k* is the number of components in the system, the subscript α denotes the parameters of the clusters of the newly-evolving phase, and β the parameters of the ambient phase where the clusters are formed. The bulk state parameters of the ambient phase are assumed to be known. The equality of the temperature and chemical potentials of the different components determine the bulk state parameters of the critical clusters. For this reason, in Equation (6), only two quantities, σ and *R*, remain unspecified.

Utilizing the surface tension as the dividing surface, the work of critical cluster formation in nucleation can be written as:

(7) $$W=\frac{1}{3}\sigma A\;,\;A=4\pi R^{2}\;,\;R=\frac{2\sigma }{p_{\alpha }-p_{\beta }}\;.$$

Consequently, σ is the only quantity one must have at one’s disposal in order to compute the size of the critical cluster, and once this quantity is specified, also the work of critical cluster formation can be determined. However, in most applications of the theory to crystal nucleation, the chemical potentials of the different components are not known, and alternative methods of computation of the pressure difference $\left(p_{\alpha }-p_{\beta }\right)$ or, equivalently, of the thermodynamic driving force [2,47,48] have to be employed.

To be definite, we consider phase formation at given external pressure, *p*, and temperature, *T* ($p=p_{\beta }$, $T=T_{\beta }$). The thermodynamic driving force of crystal nucleation can be expressed then as the change of the Gibbs free energy per unit volume of the newly-evolving phase generally as [2,25,47,48]:

(8) $$p_{\alpha }-p_{\beta }=\Delta g\left(T_{\alpha },p_{\alpha },\left\{x_{i\alpha }\right\};T_{\beta },p_{\beta },\left\{x_{i\beta }\right\}\right)\;.$$

Here, the equilibrium conditions, $T_{\alpha }=T_{\beta }=T$ (Equation (6)), have to be accounted for. These conditions eliminate in Equation (8) the temperature differences. Performing a Taylor expansion of the chemical potentials of the cluster phase in the vicinity of $p_{\alpha }=p_{\beta }=p$, we obtain:

(9) $$p_{\alpha }-p_{\beta }≅\Delta g\left(T,p\right)=\sum_{i=1}^{k}\rho_{i\alpha }\left(\mu_{i\beta }\left(T,p,\left\{x_{i\beta }\right\}\right)-\mu_{i\alpha }\left(T,p,\left\{x_{i\alpha }\right\}\right)\right)\;.$$

This approximation is quite correct provided the density of the aggregates of the newly-evolving phase depends only slightly on pressure. In addition, in such an approach, it is assumed that the composition and structure of the critical cluster are not affected by variations of pressure and temperature. The possibility to proceed in such a manner is one of the main assumptions of classical nucleation theory [2,7]. This procedure has the advantages that the knowledge of the pressure in the critical cluster and deviations of its composition and structure from the respective parameters of the evolving macroscopic phase are not required for the computations of the thermodynamic driving force.

However, also in such a formulation, the problem concerning the knowledge of the chemical potentials of the different components remains an open issue. For this reason, in crystal nucleation, where the differences of the densities of both phases (liquid and solid) are relatively small, one can replace Δg by the difference between the Gibbs free energy per unit volume of liquid and crystalline phases. In such a treatment, the thermodynamic driving force of crystal nucleation can be represented in a relatively simple form via the relation:

(10) $$\Delta g\left(T,p\right)≅\Delta h_{m}\left(\frac{T_{m}-T}{T_{m}}\right)\left[1-\gamma_{T}\left(T_{m},p_{m}\right)\frac{\left(T_{m}-T\right)}{2T_{m}}\right]+$$

$$+p_{m}\Delta v_{m}\left(\frac{p-p_{m}}{p_{m}}\right)\left[1-\gamma_{p}\left(T_{m},p_{m}\right)\frac{\left(p-p_{m}\right)}{2p_{m}}\right]\;,$$

as discussed in detail in [2,7,25,26,48]. Specific properties of the system under consideration are reflected here by the melting entropy, $\Delta s_{m}$ (or the melting enthalpy, $\Delta h_{m}=T_{m}\Delta s\left(T_{m},p_{m}\right)=T_{m}\Delta s_{m}$),

(11) $$\Delta s\left(T,p\right)=\frac{S_{liquid}\left(T,p,\left\{x_{i\beta }\right\}\right)-S_{crystal}\left(T,p,\left\{x_{i\alpha }\right\}\right)}{V_{crystal}\left(T,p,\left\{x_{i\alpha }\right\}\right)}\;,$$

the difference of the volumes between liquid and crystal phases per unit volume of the crystal:

(12) $$\Delta v\left(T,p\right)=\frac{V_{liquid}\left(T,p,\left\{x_{i\beta }\right\}\right)-V_{crystal}\left(T,p,\left\{x_{i\alpha }\right\}\right)}{V_{crystal}\left(T,p,\left\{x_{i\alpha }\right\}\right)}\;.$$

and related quantities,

(13) $$\gamma_{T}\left(T_{m},p_{m}\right)=\frac{\Delta c_{p}\left(T_{m},p_{m}\right)}{\Delta s_{m}}\;,\;\gamma_{p}\left(T_{m},p_{m}\right)=\frac{p_{m}\Delta \kappa_{T}\left(T_{m},p_{m}\right)}{\Delta v_{m}}\;.$$

Here, $c_{p}$ is the specific heat per unit volume and $\kappa_{T}$ is the isothermal compressibility [23] of the different phases, both taken at the melting or liquidus temperature, $T_{m}$, and the respective value of pressure, $p_{m}$,

(14) $$c_{p}=T{\left(\frac{\partial s}{\partial T}\right)}_{p}\;,\;\Delta c_{p}\left(T_{m},p_{m}\right)=c_{p}^{\left(liquid\right)}\left(T_{m},p_{m}\right)-c_{p}^{\left(crystal\right)}\left(T_{m},p_{m}\right)\;,$$

$$\kappa_{T}=-\frac{1}{V}{\left(\frac{\partial V}{\partial p}\right)}_{T}\;,\;\Delta \kappa_{T}\left(T_{m},p_{m}\right)=\kappa_{T}^{\left(liquid\right)}\left(T_{m},p_{m}\right)-\kappa_{T}^{\left(crystal\right)}\left(T_{m},p_{m}\right)\;.$$

In the present paper, we consider crystallization in the most frequently-occurring applications when $\Delta v_{m}>0$ holds. As a rule, the inequality, $\Delta \kappa_{T}\left(T_{m},p_{m}\right)>0$, can also to be expected to hold [2,25,49,50]. As a consequence, we obtain $\gamma_{p}\left(T_{m},p_{m}\right)>0$. This approach, utilizing Equations (10)–(14), will be employed here in the description of crystal nucleation in multi-component systems. It is important to note that it allows one by the mentioned argumentation to describe the nucleation both for stoichiometric and non-stoichiometric crystallization. The opposite case, when $\gamma_{p}\left(T_{m},p_{m}\right)<0$ holds, and the specific peculiarities arising from it will be studied in detail in an accompanying paper in an application to ice-crystal nucleation [42].

In the latter cited paper, we will treat phase formation in one-component systems. For such cases, we can avoid one of the above-mentioned approximations (replacement of Δg by the difference of the Gibbs free energy per unit volume of both phases) and directly utilize Equation (9). We get, again, in application to the crystallization of liquids:

(15) $$\Delta g\left(T,p\right)=\rho_{crystal}\left(T,p\right)\Delta \tilde{g}\left(T,p\right)\;,\;\Delta \tilde{g}\left(T,p\right)=\mu_{liquid}\left(T,p\right)-\mu_{crystal}\left(T,p\right)\;.$$

Here, $\rho_{crystal}$ is the volume density of particles in the crystal phase and μ the chemical potential per particle in the both considered phases. It is evident that in such a more correct treatment (possible only for one-component systems), the thermodynamic driving force of the transformation is essentially determined by the changes of the Gibbs free energy per particle, $\Delta \tilde{g}\left(T,p\right)$. Two methods can be employed now for further transforming Equation (15) relying on the chemical potential per particle in both phases and their pressure and temperature dependencies.

Performing a Taylor expansion of the chemical potential and accounting for:

(16) $$d\mu \left(T,p\right)=-\tilde{s}dT+\tilde{v}dp\;,$$

we get:

(17) $$\mu \left(T,p\right)=\mu \left(T_{m},p_{m}\right)+{\left.\frac{\partial \mu }{\partial T}\right|}_{T_{m},p_{m}}\left(T-T_{m}\right)+{\left.\frac{\partial \mu }{\partial p}\right|}_{T_{m},p_{m}}\left(p-p_{m}\right)$$

$$+\frac{1}{2}\left({\left.\frac{\partial^{2}\mu }{\partial T^{2}}\right|}_{T_{m},p_{m}}{\left(T-T_{m}\right)}^{2}+2{\left.\frac{\partial^{2}\mu }{\partial T\partial p}\right|}_{T_{m},p_{m}}\left(T-T_{m}\right)\left(p-p_{m}\right)+{\left.\frac{\partial^{2}\mu }{\partial p^{2}}\right|}_{T_{m},p_{m}}{\left(p-p_{m}\right)}^{2}\right)$$

or:

(18) $$\mu \left(T,p\right)=\mu \left(T_{m},p_{m}\right)-\tilde{s}\left(T_{m},p_{m}\right)\left(T-T_{m}\right)+\tilde{v}\left(T_{m},p_{m}\right)\left(p-p_{m}\right)$$

$$+\frac{1}{2}\left(-{\left.\frac{\partial \tilde{s}}{\partial T}\right|}_{T_{m},p_{m}}{\left(T-T_{m}\right)}^{2}+2{\left.\frac{\partial \tilde{v}}{\partial T}\right|}_{T_{m},p_{m}}\left(T-T_{m}\right)\left(p-p_{m}\right)+{\left.\frac{\partial \tilde{v}}{\partial p}\right|}_{T_{m},p_{m}}{\left(p-p_{m}\right)}^{2}\right)\;.$$

Here, $\tilde{s}$ is the entropy and $\tilde{v}$ the volume per particle. For the further transformation, we utilize the relations:

(19) $${\tilde{c}}_{p}=T{\left(\frac{\partial \tilde{s}}{\partial T}\right)}_{p}\;,\;{\tilde{\kappa }}_{T}=-\frac{1}{\tilde{v}}{\left(\frac{\partial \tilde{v}}{\partial p}\right)}_{T}\;,\;{\tilde{\alpha }}_{p}=\frac{1}{\tilde{v}}{\left(\frac{\partial \tilde{v}}{\partial T}\right)}_{p}\;,$$

for the specific heat, ${\tilde{c}}_{p}$, the isothermal compressibility, ${\tilde{\kappa }}_{T}$, and the isobaric thermal expansion coefficient, ${\tilde{\alpha }}_{p}$, per particle. Similar to Equation (10), we get:

(20) $$\frac{\Delta g(T,p)}{\rho_{crystal}}=\Delta {\tilde{h}}_{m}\left(\frac{T_{m}-T}{T_{m}}\right)\left[1-{\tilde{\gamma }}_{T}\frac{\left(T_{m}-T\right)}{2T_{m}}\right]+p_{m}\Delta {\tilde{v}}_{m}\left(\frac{p-p_{m}}{p_{m}}\right)\left[1-{\tilde{\gamma }}_{p}\frac{\left(p-p_{m}\right)}{2p_{m}}\right]$$

$$+{\left.\left({\left(\tilde{v}{\tilde{\alpha }}_{p}\right)}_{liquid}-{\left(\tilde{v}{\tilde{\alpha }}_{p}\right)}_{crystal}\right)\right|}_{T_{m},p_{m}}\left(T-T_{m}\right)\left(p-p_{m}\right)$$

with:

(21) $${\tilde{\gamma }}_{T}\left(T_{m},p_{m}\right)=\frac{\Delta {\tilde{c}}_{p}\left(T_{m},p_{m}\right)}{\Delta {\tilde{s}}_{m}}\;,\;{\tilde{\gamma }}_{p}\left(T_{m},p_{m}\right)=\frac{p_{m}\left({\left(\tilde{v}{\tilde{\kappa }}_{T}\right)}_{liquid}-{\left(\tilde{v}{\tilde{\kappa }}_{T}\right)}_{crystal}\right)}{\Delta {\tilde{v}}_{m}}$$

and:

(22) $$\Delta {\tilde{s}}_{m}={\tilde{s}}_{liquid}\left(T_{m},p_{m}\right)-{\tilde{s}}_{crystal}\left(T_{m},p_{m}\right)\;,\;\Delta {\tilde{v}}_{m}={\tilde{v}}_{liquid}\left(T_{m},p_{m}\right)-{\tilde{v}}_{crystal}\left(T_{m},p_{m}\right)\;.$$

The terms containing the product of pressure and temperature differences vanish if the volumes per particle in the melt and the crystal are assumed to be equal, as done in the derivation of Equation (10).

Alternatively, starting directly with Equation (16), we may write:

(23) $$\mu \left(T,p\right)=\mu \left(T_{m},p_{m}\right)-\int_{T_{m}}^{T}\tilde{s}\left(T,p_{m}\right)dT+\int_{p_{m}}^{p}\tilde{v}\left(T,p\right)dp$$

resulting in:

(24) $$\mu_{liquid}\left(T,p\right)-\mu_{crystal}\left(T,p\right)=-\int_{T_{m}}^{T}\Delta \tilde{s}\left(T,p_{m}\right)dT+\int_{p_{m}}^{p}\Delta \tilde{v}\left(T_{m},p\right)dp\;.$$

Here, Δs and Δv are defined by Equation (22), again. We introduced here in addition the approximation $\Delta \tilde{v}\left(T,p\right)≅\Delta \tilde{v}\left(T_{m},p\right)$. With a truncated Taylor expansion of $\Delta \tilde{s}\left(T,p_{m}\right)$ and $\Delta \tilde{v}\left(T,p_{m}\right)$:

(25) $$\Delta \tilde{s}\left(T,p_{m}\right)≅\Delta \tilde{s}\left(T_{m},p_{m}\right)+{\left.\frac{\partial \Delta \tilde{s}}{\partial T}\right|}_{T_{m},p_{m}}\left(T-T_{m}\right)=\Delta {\tilde{s}}_{m}+\Delta {\tilde{c}}_{p}\left(T_{m},p_{m}\right)\frac{\left(T-T_{m}\right)}{T_{m}}\;,$$

(26) $$\Delta {\tilde{v}}_{m}\left(T_{m},p\right)≅\Delta {\tilde{v}}_{m}+\left({\left(\tilde{v}{\tilde{\kappa }}_{T}\right)}_{liquid}-{\left(\tilde{v}{\tilde{\kappa }}_{T}\right)}_{crystal}\right)\left(p-p_{m}\right)\;,$$

Equation (24) yields:

(27) $$\Delta \tilde{g}\left(T,p\right)=\Delta {\tilde{h}}_{m}\left(\frac{T_{m}-T}{T_{m}}\right)\left[1-{\tilde{\gamma }}_{T}\frac{\left(T_{m}-T\right)}{2T_{m}}\right]+p_{m}\Delta {\tilde{v}}_{m}\left(\frac{p-p_{m}}{p_{m}}\right)\left[1-{\tilde{\gamma }}_{p}\frac{\left(p-p_{m}\right)}{2p_{m}}\right]\;,$$

again. Note that the absence of terms proportional to the product of pressure and temperature differences occurring in Equation (20) is due to the approximation $\Delta \tilde{v}\left(T,p\right)≅\Delta \tilde{v}\left(T_{m},p\right)$ utilized here in the derivations.

### 2.2. Temperature and Pressure Dependence of the Surface Tension

As is evident from the Gibbs adsorption equation in its general form ([1], see Equation (43) somewhat later) and its consequences, the superficial (or surface) entropy is one of the basic parameters determining the dependence of the surface tension on the thermodynamic state parameters of two phases in thermodynamic equilibrium. A comprehensive analysis of this circle of problems was performed, for example, by Rusanov [46], correlating the superficial quantities with directly-measurable changes of the surface properties including the surface tension. However, the values of the superficial quantities cannot be determined from purely thermodynamic considerations, and additional model assumptions have to be introduced in order to arrive at quantitative results.

Advancing particular models of the solid–liquid interface, Spaepen [51,52,53] developed several approaches for the determination of the surface tension and its effect on crystal nucleation and growth. He underlined in his studies the entropic origin of the surface tension correlating its value with the entropy loss in the ordering of the liquid, suggested as a possible explanation already earlier by Turnbull [54]. In the present paper, we will outline basic relations for the temperature and pressure dependence of the surface tension for both one- and multi-component systems, determining it exclusively via the change of the bulk contributions of the entropy, the melting entropy, in the respective phase transformations. Since melting is essentially characterized by the loss of ordering, these ideas are also reflected in the treatment presented here. Our approach allows us to obtain the dependencies of the surface tension of planar interfaces and critical clusters on pressure and temperature, correlating it with directly-measurable thermodynamic quantities. The results will be employed then for the determination of the Tolman parameter and related quantities.

For the first time, the relation between surface tension and the change of entropy in phase transformations was intensively analyzed by Josef Stefan [43] in the application to vapor condensation. Stefan noted in his study that a certain work is required to transfer a particle (atom, molecule) from the bulk to the surface of a liquid. The same work he supposed has to be performed in order to transfer it from the surface to the vapor phase (in German: *“Die Vergrösserung der Oberfläche der Flüssigkeit um den Querschnitt eines Moleküls erfordert denselben Aufwand an Energie, wie die Verdampfung eines Moleküls.”* [43]). This statement clearly reflects his point of view concerning the intrinsic correlation between capillarity and the heat of evaporation. Stefan also noted already that the work one has to perform in such processes is different for planar, concave, and convex interfaces expressing implicitly already the ideas of a curvature dependence of the specific surface energy. In addition, he discussed different requirements for which the relation between specific surface energy and entropy of the phase transition can be expected to hold. In particular, he underlined the necessity that the particles have to interact via short-range interaction forces and that the density of the liquid has to be the same in the bulk and near the surface. Assuming a continuous change of density from the liquid bulk values to the vapor, then he believed these considerations to be no longer valid. Further, it is required according to Stefan that the molecules of the vapor be of the same kind as the molecules of the liquid and not undergo during the phase formation process any kind of transformation.

In application to crystal nucleation, these ideas have been advanced, in particular, by Skapski [55,56] and Turnbull [14,54]. The resulting expression for the surface tension is conventionally denoted as the Skapski–Turnbull relation and expressed as [2,24]:

(28) $$\sigma \left(T_{m},p_{m}\right)=\xi \frac{q_{m}\left(T_{m},p_{m}\right)}{N_{A}^{1/3}v_{m}^{2/3}\left(T_{m},p_{m}\right)}$$

or:

(29) $$\sigma \left(T_{m},p_{m}\right)=\xi \frac{\tilde{q}\left(T_{m},p_{m}\right)}{{\tilde{v}}^{2/3}\left(T_{m},p_{m}\right)}\;.$$

Here, $q_{m}$ and $v_{m}$ are the molar heat of melting and the molar volume and $\tilde{q}$ and $\tilde{v}$ are the respective values per particle of the liquid (in condensation and boiling), respectively, the crystal (in crystallization), and $N_{A}$ is the Avogadro number. This relation is frequently applied to the specification of the surface tension for crystal-liquid equilibrium at planar interfaces.

In order to generalize this relation to non-equilibrium states and to the description of critical clusters, we replaced the heat of melting by the change of enthalpy. In terms of the generalized Gibbs’ approach [2,3,4,7], then expressions for the change of enthalpy in cluster formation have been developed for clusters not being in equilibrium with the ambient phase. As shown by us in [24,25], in general, the change of the enthalpy, $\Delta \tilde{h}$, if one particle in a one-component system is transferred from the cluster (specified by α) to the surrounding ambient phase (specified by β), is given by:

(30) $$\Delta \tilde{h}=T_{\beta }{\tilde{s}}_{\beta }\left(T_{\beta },p_{\beta }\right)-T_{\alpha }{\tilde{s}}_{\alpha }\left(T_{\alpha },p_{\alpha }\right)+\left[\mu_{\beta }\left(T_{\beta },p_{\beta }\right)-\mu_{\alpha }\left(T_{\alpha },p_{\alpha }\right)\right]\;.$$

Utilizing the Gibbs equilibrium conditions (equality of temperature, $T_{\alpha }=T_{\beta }=T$, and of chemical potentials, $\mu_{\alpha }=\mu_{\beta }$) and omitting the subscript β for the pressure of the ambient phase ($p_{\beta }=p$), the change of enthalpy is reduced to differences of entropy per particle, $\tilde{s}$, in the different phases, i.e.:

(31) $$\Delta \tilde{h}=T\left({\tilde{s}}_{\beta }\left(T,p\right)-{\tilde{s}}_{\alpha }\left(T,p_{\alpha }\right)\right)\;.$$

The latter relation holds both for equilibrium phase coexistence at planar interfaces (with a value of the surface tension equal to $\sigma =\sigma_{\infty }$) and for critical clusters (with a value of the surface tension equal to σ). In the application to one-component systems, we may write, consequently,

(32) $$\frac{\sigma (T,p)}{\sigma (T_{m},p_{m})}=\frac{T\Delta \tilde{s}\left(T,p\right)}{T_{m}\Delta \tilde{s}\left(T_{m},p_{m}\right)}\;.$$

In the latter relation, we made again the approximation $p_{\alpha }≅p$ conventionally utilized in CNT. Note that considering here the ratio $\sigma \left(T,p\right)/\sigma \left(T_{m},p_{m}\right)$, we avoid uncertainties connected with the specification of the value of the fit parameter ξ in Equations (28) and (29). It is assumed here further that this parameter only slightly depends on the degree of supersaturation (see [24] for a more detailed discussion).

Performing a Taylor expansion of $\Delta \tilde{s}\left(T,p\right)$ in the vicinity of $\left(T_{m},p_{m}\right)$, we arrive at:

(33) $$\Delta \tilde{s}\left(T,p\right)=\Delta \tilde{s}\left(T_{m},p_{m}\right)+{\left(\frac{\partial \Delta \tilde{s}\left(T,p\right)}{\partial T}\right)}_{T_{m},p_{m}}\left(T-T_{m}\right)+{\left(\frac{\partial \Delta \tilde{s}\left(T,p\right)}{\partial p}\right)}_{T_{m},p_{m}}\left(p-p_{m}\right)+\ldots$$

With:

(34) $${\tilde{c}}_{p}=T{\left(\frac{\partial \tilde{s}}{\partial T}\right)}_{p}\;,\;{\left(\frac{\partial \tilde{s}}{\partial p}\right)}_{T}=-{\left(\frac{\partial \tilde{v}}{\partial T}\right)}_{p}\;,\;{\tilde{\alpha }}_{p}=\frac{1}{\tilde{v}}{\left(\frac{\partial \tilde{v}}{\partial T}\right)}_{p}\;,$$

we obtain:

(35) $$\frac{\sigma (T,p)}{\sigma (T_{m},p_{m})}≅\frac{T}{T_{m}}\left(1-\frac{\Delta {\tilde{c}}_{p}\left(T_{m},p_{m}\right)}{\Delta {\tilde{s}}_{m}}\frac{T_{m}-T}{T_{m}}-\frac{\Delta \left[\tilde{v}\left(T_{m},p_{m}\right){\tilde{\alpha }}_{p}\left(T_{m},p_{m}\right)\right]}{\Delta {\tilde{s}}_{m}}\left(p-p_{m}\right)\right)\;,$$

where:

(36) $$\Delta \left[\tilde{v}\left(T_{m},p_{m}\right){\tilde{\alpha }}_{p}\left(T_{m},p_{m}\right)\right]={\tilde{v}}^{\left(liquid\right)}\left(T_{m},p_{m}\right){\tilde{\alpha }}_{p}^{\left(liquid\right)}\left(T_{m},p_{m}\right)$$

$$-{\tilde{v}}^{\left(crystal\right)}\left(T_{m},p_{m}\right){\tilde{\alpha }}_{p}^{\left(crystal\right)}\left(T_{m},p_{m}\right)\;.$$

Here, ${\tilde{\alpha }}_{p}$ is the isobaric thermal expansion coefficient. Assuming near identity of the volume per particle in the liquid and the crystal (${\tilde{v}}^{\left(crystal\right)}≅{\tilde{v}}^{\left(liquid\right)}≅\tilde{v}$), we obtain approximately:

(37) $$\frac{\sigma (T,p)}{\sigma (T_{m},p_{m})}≅\frac{T}{T_{m}}\left(1-\frac{\Delta {\tilde{c}}_{p}\left(T_{m},p_{m}\right)}{\Delta {\tilde{s}}_{m}}\frac{T_{m}-T}{T_{m}}-\frac{\Delta {\tilde{\alpha }}_{p}\left(T_{m},p_{m}\right)}{\Delta {\tilde{s}}_{m}}\left(p-p_{m}\right)\tilde{v}\left(T_{m},p_{m}\right)\right)\;.$$

In order to extend these relations to multi-component systems, we replace in Equation (32) entropy changes per particle via enthalpy, respectively entropy changes per unit volume. Generally, the entropy of a certain amount of a given phase can be expressed as a function of temperature, pressure, and the number of particles of the different components:

(38) $$S=S(T,p,n_{1},n_{2},\ldots ,n_{k})\;.$$

Using Euler’s homogeneous function theorem, we obtain:

(39) $$S=S\left(T,p,n_{1},n_{2},\ldots ,n_{k}\right)=\sum_{i=1}^{k}s_{i}n_{i}\;,\;s_{i}={\left(\frac{\partial S}{\partial n_{i}}\right)}_{T,p,n_{j,j\neq 1}}\;.$$

In line with the basic assumption of classical nucleation theory commonly employed in the determination of the thermodynamic driving force (see Equations (10)–(14)), we assume that the change of temperature and/or pressure does not affect the composition and/or structure of the critical clusters. The difference of the entropy per unit volume of the newly-evolving phase between liquid and crystal is given then in analogy to Equation (9) as:

(40) $$\Delta s\left(T,p\right)=\sum_{i=1}^{k}\rho_{i\alpha }\left(s_{i\beta }\left(T,p,\left\{x_{i\beta }\right\}\right)-s_{i\alpha }\left(T,p,\left\{x_{i\alpha }\right\}\right)\right)\;.$$

Similar as in the determination of the thermodynamic driving force in crystal nucleation, where the differences of the densities of the both phases (liquid and solid) are relatively small, we replace in this way Δs by the difference between the entropies per unit volume of liquid and crystalline phases. The dependence of the surface tension on pressure and temperature as derived in [24,25,26] can be expressed then in the form:

(41) $$\frac{\sigma (T,p)}{\sigma (T_{m},p_{m})}≅\frac{T}{T_{m}}\left(1-\gamma_{T}\left(T_{m},p_{m}\right)\frac{T_{m}-T}{T_{m}}-\frac{\Delta \alpha_{p}\left(T_{m},p_{m}\right)}{\Delta s_{m}}\left(p-p_{m}\right)\right)\;,$$

where:

(42) $$\alpha_{p}=\frac{1}{V}{\left(\frac{\partial V}{\partial T}\right)}_{p}\;,\;\Delta \alpha_{p}\left(T_{m},p_{m}\right)=\alpha_{T}^{\left(liquid\right)}\left(T_{m},p_{m}\right)-\alpha_{T}^{\left(crystal\right)}\left(T_{m},p_{m}\right)\;,$$

again. In Equation (41), Δs and $\Delta c_{p}$ are the differences between the entropy and the specific heat per unit volume of the liquid and the crystal, again, and $\Delta \alpha_{p}$ the difference between the isobaric thermal expansion coefficients of both phases. Having in mind the differences in the meaning of the entropy differences in Equations (37) and (41), both relations are widely equivalent.

### 2.3. Tolman Equation and Tolman Parameter

For the derivation of the Tolman equation and the specification of the Tolman parameter, in addition to the equilibrium conditions, the Gibbs adsorption equation is required. It reads in the general form [1,8,46,47]:

(43) $$S_{\sigma }dT+Ad\sigma +\sum_{i=1}^{k}n_{i\sigma }d\mu_{i}=0\;.$$

Here, *A* is the surface area of a given surface element, and $S_{\sigma }$ and $n_{i\sigma }$ are the respective values of the so-called superficial entropy and particle numbers assigned in the framework of Gibbs’ theory of surface phenomena formally to the interface treated to be of zero thickness.

Gibbs [1] and also Tolman [11] considered phase formation in one-component fluids at some given temperature changing the degree of deviation from equilibrium by variations of the pressure, $p=p_{\beta }$, of the ambient phase. At such conditions, Equations (6) and (43) yield:

(44) $$\mu_{\alpha }\left(T,p_{\alpha }\right)=\mu_{\beta }\left(T,p_{\beta }\right)\;,\;p_{\alpha }-p_{\beta }=\frac{2\sigma }{R}\;,$$

(45) $$Ad\sigma +n_{\sigma }d\mu_{\beta }=0\;\mathrm{or}\;Ad\sigma +\left(n_{\sigma }{\tilde{v}}_{\beta }\right)dp_{\beta }=0\;.$$

Taking the differential of the two relations in Equation (44) accounting for the constancy of temperature, we get:

(46) $${\tilde{v}}_{\alpha }dp_{\alpha }={\tilde{v}}_{\beta }dp_{\beta }\;,\;dp_{\alpha }-dp_{\beta }=d\left(\frac{2\sigma }{R}\right)\;.$$

After some straightforward transformations of Equations (45) and (46), we obtain Equation (1) with the parameter δ given by:

(47) $$\delta =\delta_{k=1}^{\left(p\right)}=\frac{\Gamma }{\rho_{\alpha }-\rho_{\beta }}\;,\;\Gamma =\frac{n_{\sigma }}{A}\;.$$

In the above relations, $\tilde{v}$ is the volume per particle, and $\rho =1/\tilde{v}$ is the volume density of particles. This expression for δ can then be reformulated in the form of Equation (2) as done by Tolman in [11]. The indices in $\delta_{k=1}^{\left(p\right)}$ specify that this value of the Tolman parameter is obtained for phase formation in one-component systems (k=1) at isothermal conditions, when the pressure, $p=p_{\beta }$, is varied.

Alternatively, we may vary the temperature ($T=T_{\alpha }=T_{\beta }$), leaving the pressure of the ambient phase, $p_{\beta }$, unchanged. For this case, Equations (6) and (43) lead to the following relations:

(48) $${\tilde{v}}_{\alpha }dp_{\alpha }=\left({\tilde{s}}_{\alpha }-{\tilde{s}}_{\beta }\right)dT\;,\;dp_{\alpha }=d\left(\frac{2\sigma }{R}\right)\;,$$

and:

(49) $$S_{\sigma }dT+Ad\sigma +n_{\sigma }d\mu_{\beta }\left(T,p_{\beta }\right)=0$$

or:

(50) $$\left[\left(S_{\sigma }/A\right)-\left(n_{\sigma }/A\right){\tilde{s}}_{\beta }\right]dT+d\sigma =0\;,$$

respectively. Here, $\tilde{s}$ is the entropy per particle in the bulk of both phases, correspondingly. A combination of Equations (48) and (50) results in Equation (1), again, but this time with the value of δ equal to:

(51) $$\delta =\delta_{k=1}^{\left(T\right)}=\frac{{\tilde{v}}_{\alpha }\left[\left(S_{\sigma }/A\right)-\left(n_{\sigma }/A\right){\tilde{s}}_{\beta }\right]}{{\tilde{s}}_{\alpha }-{\tilde{s}}_{\beta }}\;.$$

The indices in $\delta_{k=1}^{\left(T\right)}$ specify, again, that this value of the Tolman parameter is obtained for phase formation in one-component systems (k=1) at isobaric conditions, when the temperature, *T*, is varied. Taking $\delta =\delta \left(R\to \infty \right)=\delta_{\infty }$ as constant, for both cases of either temperature or pressure-induced phase formation in one-component systems, the curvature dependence of the surface tension is described by Tolman’s approximative relation, Equation (3), but with different values of the parameter $\delta_{\infty }$, $\delta_{\infty }=\lim_{R\to \infty }\delta_{k=1}^{\left(p\right)}$ (Equation (47) and $\delta_{\infty }=\lim_{R\to \infty }\delta_{k=1}^{\left(T\right)}$ (Equation (51), for the both considered methods of the variation of the degree of deviation from thermodynamic equilibrium.

Allowing for variations of both pressure of the ambient phase, $p_{\beta }$, and temperature, *T* (accounting for $T_{\alpha }=T_{\beta }=T$), we obtain the following set of equations:

(52) $${\tilde{v}}_{\alpha }dp_{\alpha }-{\tilde{s}}_{\alpha }dT={\tilde{v}}_{\beta }dp_{\beta }-{\tilde{s}}_{\beta }dT\;,\;dp_{\alpha }-dp_{\beta }=d\left(\frac{2\sigma }{R}\right)\;,$$

(53) $$\left[\left(S_{\sigma }/A\right)-\left(n_{\sigma }/A\right){\tilde{s}}_{\beta }\right]dT+\left(\Gamma {\tilde{v}}_{\beta }\right)dp_{\beta }+d\sigma =0\;.$$

Eliminating the pressure of the critical cluster via Equations (52), we obtain a relation correlating infinitesimal changes of σ, $p_{\beta }$, *T*, and *R*. Accounting in addition for Equation (53), the surface tension can be considered in such a case as a function of pressure and temperature ($\sigma =\sigma (p_{\beta },T)$), pressure and critical cluster size ($\sigma =\sigma (p_{\beta },R)$), or temperature and critical cluster size (σ=σ(T,R)). A reduction to Equation (1) and a Tolman-like expression, σ=σ(R), is impossible at such conditions.

Varying only pressure or temperature and keeping the composition of the ambient phase fixed, similar results as derived here for one-component systems can be obtained also for multi-component systems. Indeed, for a *k*-component system, the conditions of the equality of temperature and chemical potentials yield:

(54) $$\mu_{i\alpha }\left(T,p_{\alpha },x_{1,\alpha },x_{2,\alpha },\ldots ,x_{k-1,\alpha }\right)=\mu_{i\beta }\left(T,p_{\beta },x_{1,\beta },x_{2,\beta },\ldots ,x_{k-1,\beta }\right),\;i=1,2,\ldots ,k\;.$$

At fixed values of temperature, this set of equations determines the bulk state parameters of the cluster ($p_{\alpha },x_{1,\alpha },x_{2,\alpha },\ldots ,x_{k-1,\alpha }$) as a function of the state parameters of the ambient phase. We assume here first that, at fixed values of temperature, the degree of deviation from equilibrium is determined by variations of pressure keeping the composition of the melt unchanged. In classical nucleation theory in the application to crystal nucleation, it is supposed that such changes do not affect the composition of the critical clusters. Utilizing also the latter assumption, Equation (54) supplies us with a linear relation $dp_{\alpha }=\gamma_{1}^{\left(p\right)}dp_{\beta }$ similar to the first term in Equation (46). With $\mu_{i}=\mu_{i\beta }$ (as always employed also in the analysis of the one-component case), the Gibbs adsorption isotherm (Equation (43) with *T* = constant) yields:

(55) $$d\sigma +dp_{\beta }\sum_{i=1}^{k}\left(\frac{n_{i\sigma }}{A}\right)\frac{\partial \mu_{i\beta }}{\partial p_{\beta }}=0$$

or, similar to Equation (45), $d\sigma +\gamma_{2}^{\left(p\right)}dp_{\beta }=0$. A substitution of the relations $dp_{\alpha }=\gamma_{1}^{\left(p\right)}dp_{\beta }$ and $d\sigma +\gamma_{2}^{\left(p\right)}dp_{\beta }=0$ into the relation for pressure equilibrium in the differential form (the second relation in Equation (46)) results in Equation (1), again, with:

(56) $$\delta =\delta_{k}^{\left(p\right)}=\frac{\gamma_{2}^{\left(p\right)}}{\gamma_{1}^{\left(p\right)}-1}\;.$$

Keeping now, again, the pressure constant and varying the temperature, the equilibrium conditions, Equation (54), determine the bulk state parameters of the cluster phase in dependence on the temperature and molar fractions of the liquid. Since the latter parameters are fixed and once the composition of the newly-evolving phase is assumed again to be not affected by such variations, we obtain $dp_{\alpha }=\gamma_{1}^{\left(T\right)}dT$. The Gibbs adsorption equation, Equation (43), yields then:

(57) $$\left(S_{\sigma }/A\right)dT+d\sigma +dT\sum_{i=1}^{k}\left(\frac{n_{i\sigma }}{A}\right)\frac{\partial \mu_{i\beta }}{\partial T}=0\;.$$

This relation can be abbreviated as $d\sigma +\gamma_{2}^{\left(T\right)}dT=0$. With the conditions for pressure equilibrium in differential form (the second relation in Equation (48)), we obtain Equation (1), again, this time with:

(58) $$\delta =\delta_{k}^{\left(T\right)}=\frac{\gamma_{2}^{\left(T\right)}}{\gamma_{1}^{\left(T\right)}}\;.$$

In cases when both the temperature ($T=T_{\alpha }=T_{\beta }$) and pressure, $p_{\beta }$, of the liquid are varied, the curvature dependence of the surface tension is determined by a combination of the above derived equations. We obtain then:

(59) $$dp_{\alpha }=\gamma_{1}^{\left(p\right)}dp_{\beta }+\gamma_{1}^{\left(T\right)}dT\;,$$

(60) $$dp_{\alpha }-dp_{\beta }=d\left(\frac{2\sigma }{R}\right)\;.$$

(61) $$d\sigma +\gamma_{2}^{\left(p\right)}dp_{\beta }+\left(\left(S_{\sigma }/A\right)+\gamma_{2}^{\left(T\right)}\right)dT=0\;,$$

Equations (59) and (60) result similarly to the one-component case in a relation correlating infinitesimal changes of σ, $p_{\beta }$, *T*, and *R*. Consequently, Equations (59)–(61) cannot be reduced, again, to Tolman-type expressions for the curvature dependence of the surface tension (see also [15]) even if, as done here, the composition of the newly-evolving phase is assumed to be not affected by variations of external pressure and temperature. The situation becomes even more complex if a possible variation of the composition and/or structure of the critical cluster phase in response to variations of pressure and temperature is accounted for. Consequently, we may conclude that the curvature dependence of the surface tension of critical clusters in multi-component systems can be reduced to Tolman-like expressions only in cases when either the temperature or pressure of the ambient phase is varied, provided that the composition of both the ambient and the newly-evolving phases is unchanged.

## 3. Application to Crystal Nucleation

### 3.1. Peculiarities of the Application of the Tolman Equation to Crystal Nucleation

The theoretical description of the thermodynamic aspects of crystal nucleation is confronted with several serious problems. One of them consists of the specification of the thermodynamic driving force of crystal nucleation [2,7,48]. Here, it is commonly assumed that the thermodynamic driving force for crystal nucleation can be determined as the difference of the Gibbs free energies of macroscopic samples of liquid and crystal at the same values of pressure and temperature. This is, as already discussed here, a good approximation as far as density, composition, and structure of the critical crystal clusters are not affected by variations of external pressure or temperature [7,48]. The second main problem is that the surface tension even for the planar equilibrium coexistence of liquid and crystal cannot be measured as a rule with the accuracy required for the description of nucleation. For this reason, the surface tension is taken commonly as a fit parameter [2,30,57]. Utilizing, in such a treatment, the capillarity approximation, such an approach results in serious problems in the theoretical description of nucleation in terms of CNT as discussed in detail in [32,57]. An account of the size dependence of the surface tension is, consequently, absolutely necessary in classical nucleation theory in order to avoid this.

Employing classical nucleation theory, one more complication occurs. Following the basic assumptions of CNT (critical crystallites governing nucleation are treated as small objects having widely the same properties as the respective macroscopic samples), critical crystal clusters are not spheres, but have a shape determined by the Gibbs–Curie–Wulff theorem [1,58,59,60,61]. Utilizing such a more correct description would lead to a considerable increase of the number of unknown parameters remove in advance, the values of the surface tension for the different crystal faces. For this reason, in most applications of CNT to the interpretation of experimental data, a simplified description is used assuming a spherical shape of the critical clusters with a radius, *R* [2,57]. In order to proceed in such a way, a theoretical foundation is required. Here, we demonstrate by two different methods how such a simplification of the description can be theoretically founded (see also [15]).

As advanced in detail in [1,58,59,60,61,62,63], accounting for the Gibbs–Curie–Wulff theorem, the work of critical crystal cluster formation, *W*, can be expressed, in general, as:

(62) $$W=\frac{1}{3}\int \sigma \left(A\right)dA=\frac{1}{3}\sum_{i}\sigma_{i}A_{i}\;.$$

In the first term in the above equation, the integration has to be performed over the surface of the crystallite with values of the surface tension depending on the point of the surface considered. In the second term, $\sigma_{i}$ are the values of the surface tension for the different crystal faces with the surface areas $A_{i}$. The above relation can be simplified by introducing an effective surface energy, σ, defining it as:

(63) $$\sigma =\frac{1}{4\pi R^{2}}\int \sigma \left(A\right)dA=\frac{1}{4\pi R^{2}}\sum_{i}\sigma_{i}A_{i}\;.$$

Here, *R* is the radius of a sphere having the same volume as the equilibrium crystallite with a shape determined by the Gibbs–Curie–Wulff theorem. The work of critical cluster formation for a critical cluster of spherical shape where the radius is defined via the surface tension is then given by:

(64) $$W=\frac{1}{3}\sigma A\;,\;A=4\pi R^{2}\;.$$

Similar conclusions one can be derived even in a simpler way not relying on the Gibbs–Curie–Wulff theorem. We only employ the properties of the critical clusters determined by Gibbs’ equilibrium conditions, Equation (6), assuming the critical clusters to be of a spherical shape. For any given value of the work of critical cluster formation governing nucleation in the system under consideration, Equation (64) supplies us with an additional equation for the determination of σ and *R* in terms of the simplified model. Consequently, knowing the value of the work of critical cluster formation, *W*, the Young–Laplace equation, $p_{\alpha }-p_{\beta }=2\sigma /R$, determines uniquely both the size of the critical cluster and the value of its surface tension. Consequently, whatever the shape of a real critical crystal cluster is, one can always describe it by a simplified model of a sphere with the well-defined above considerations, values of the radius, and the surface tension.

The above considerations are closely connected with another problem in applying the Gibbs–Tolman approach to the curvature dependence of critical clusters to small crystallites. The Gibbs–Tolman approach is developed for fluid–fluid interfaces. The basic equations for the description of small crystallites are more complex, and also, the equilibrium conditions have to be modified [1,64], involving a variety of additional and even partly-unresolved questions. For example, in the Young–Laplace equation, the surface tension has to be replaced at certain conditions by the surface stress [64]. These more advanced relations result in Equation (62) as shown first by Gibbs [1]. However, in the overwhelming majority of theoretical interpretations of crystal nucleation experiments [2,5,14,30,31,61,62,63,65], Equations (6)–(9) are taken as the basis of the analysis. For this reason, it is also of great interest to analyze how curvature effects on the surface properties of small crystallites can be incorporated in such a treatment. This approach is also followed in the applications of the Gibbs–Tolman approach to crystallization, as we will discuss below. Going over from the Gibbs–Curie–Wulff construction to a description in terms of a critical cluster of radius *R* with an effective average value of the surface tension, one can assume that for this simplified description, Gibbs’ basic relations for fluid–fluid interfaces hold. Utilizing these relations, we will analyze here the problem of how the curvature dependence of the surface tension can be described in such a model approach in the application to crystallization provided only one specific property of the critical crystallites is accounted for, the dependence of the work of their formation on pressure and/or temperature.

Having given the foundation in this way of such a simplified treatment in crystal nucleation, we can employ directly the results obtained for the description of the curvature dependence of the surface tension as derived in Section 2. In this section, the conditions are specified at which conditions the Tolman equation can be employed for the description of the curvature dependence of the surface tension. Crystallization has been mainly studied so far by varying the temperature at constant external pressure [2,57]. However, this is not the case studied by Tolman. Indeed, Tolman noted in his paper [11]: *“We shall be concerned with the effect of changes in radius on surface tension in the case of droplets and vapor composed of a single substance maintained at some given constant temperature”*. Consequently, any reference to Tolman in applying his relation to the study of crystal nucleation at constant pressure varying temperature is, without performing a check of its validity, incorrect. Anyway, it can be done, as demonstrated by us first in [15] and reviewed briefly here in Section 2, since also for this case, the curvature dependence of the surface tension can be described by identical relations, however with another definition of the Tolman parameter. As shown there as well, the Tolman equation can by utilized for the description of such dependence even for phase formation in multi-component systems provided the composition of both the liquid and the crystal phases does not change when either external pressure or temperature are varied.

### 3.2. Tolman Equation and Its Generalization in the Application to Crystallization: The Tolman Parameter

A method to determine the Tolman parameter to describe the curvature dependence of the surface tension for critical crystals in multi-component liquids for crystallization caused by variations of either temperature or pressure was developed first in [15]. It involves basically Equation (41) derived here earlier. Moreover, the radius, *R*, of the critical cluster (referring to the surface tension, again) can be expressed at the mentioned conditions in a good approximation as [2,7,25]:

(65) $$R=\frac{2\sigma }{\Delta g(T,p)}\;.$$

Here, Δg is the difference of Gibbs’ free energy per unit volume between liquid and crystal, both taken at the same pressure and temperature, and σ is the surface tension for the chosen dividing surface. For the considered case of small deviations from equilibrium, the thermodynamic driving force as a function of undercooling is given by the Tammann–Meissner–Rie equation [6,48]:

(66) $$\Delta g\left(T\right)≅\Delta h_{m}\left(\frac{T_{m}-T}{T_{m}}\right)\;,\;\Delta h_{m}=\Delta h\left(T_{m},p_{m}\right)=T_{m}\Delta s_{m}\;.$$

Here, $\Delta h_{m}>0$ is the melting enthalpy per unit volume of the crystal phase and $\Delta s_{m}$ the respective melting entropy. Similarly, we can write for pressure-induced nucleation [25]:

(67) $$\Delta g\left(p\right)≅p_{m}\Delta v_{m}\left(\frac{p-p_{m}}{p_{m}}\right)\;,$$

$$\Delta v_{m}=\Delta v\left(T_{m},p_{m}\right)\;,\;\Delta v\left(T,p\right)=\frac{V_{l}\left(T,p,\left\{x_{il}\right\}\right)-V_{c}\left(T,p,\left\{x_{ic}\right\}\right)}{V_{c}\left(T,p,\left\{x_{ic}\right\}\right)}\;,$$

where $V_{l}$ and $V_{c}$ are the volumes of a certain amount of the material in the liquid (l) and crystalline (c) states.

To determine the Tolman parameter, $\delta =\delta_{\infty }=\delta \left(R\to \infty \right)$, we rewrite Equation (3) in the form:

(68) $$\delta_{\infty }=\lim_{R\to \infty }\frac{R}{2}\left(\frac{\sigma_{\infty }}{\sigma }-1\right)=\lim_{R\to \infty }\frac{R}{2\sigma }\sigma_{\infty }\left(1-\frac{\sigma }{\sigma_{\infty }}\right)=\lim_{R\to \infty }\frac{\sigma_{\infty }}{\Delta g}\left(1-\frac{\sigma }{\sigma_{\infty }}\right)\;,$$

and employ Equations (41)–(67) for the further transformations. At a decrease of temperature, *T*, at constant pressure, $p=p_{m}$, we arrive at:

(69) $$\delta_{\infty }^{\left(T\right)}≅\sigma_{\infty }\frac{1+\gamma_{T}\left(T_{m},p_{m}\right)}{\Delta h_{m}}\;\mathrm{at}\;p=p_{m}\;.$$

Varying pressure, *p*, at constant temperature, $T=T_{m}$, we obtain instead the relation:

(70) $$\delta_{\infty }^{\left(p\right)}≅\sigma_{\infty }\frac{\Delta \alpha_{p}\left(T_{m},p_{m}\right)}{\Delta v_{m}\Delta s_{m}}=\sigma_{\infty }\frac{T_{m}\Delta \alpha_{p}\left(T_{m},p_{m}\right)}{\Delta v_{m}\Delta h_{m}}\;\mathrm{at}\;T=T_{m}\;.$$

In both cases, the Tolman parameter is determined by the surface tension for a planar equilibrium liquid–crystal interface and a combination of bulk properties of liquid and crystal in such states.

The specification of the Tolman parameter can be further advanced by determining the parameter $l_{\infty }$ in Equations (4) and (5). The starting point is the following: As shown by Skripov and Baidakov based on a thorough analysis of experimental data first in [66] and reconfirmed in [67], there does not exist a spinodal in one-component melt crystallization in undercooled liquids. In [26], some of us showed that both in pressure- and temperature-induced crystallization, a spinodal does not occur also in multi-component liquids as far as the basic assumption of CNT is fulfilled, i.e., that the composition and/or structure of the critical clusters are not affected by the variations of pressure and temperature. For this reason, in the application to melt crystallization, correction terms to the Tolman parameter cannot be advanced relying on specific properties of the spinodal curve, as will be done here later in the application to condensation and boiling. However, for both temperature- and pressure-induced crystallization, there exists a well-defined maximum of the steady-state nucleation rate in dependence on either temperature or pressure. This basic feature can be employed to advance a more precise specification of the Tolman parameter, treating it in a more general manner as compared to its original meaning (see Equation (5) and its analysis). Details of such an approach are given in [15].

Finally, we would like to remind that for large degrees of undercooling, additional terms have to be accounted for in our relations, modifying possibly the above derived general conclusions for that range of supersaturations (see also [24]). One of these factors is the effect of different values of the pressure in the cluster phase as compared to the surrounding liquid; other factors which may affect the value of the surface tension are connected with possible changes of the composition and/or structure of the critical clusters in dependence on the variations of pressure and temperature. In line with the basic assumptions of CNT, the latter effects are completely neglected here. We will return briefly to this topic comparing our theoretical results with experimental data.

### 3.3. Brief Comparison with Experimental Data and Computer Simulation Studies

In our approach, the increase of the degree of deviation from equilibrium (both via the decrease of temperature, ${\left(d\sigma /dT\right)}_{p}>0$, and the increase of pressure, ${\left(d\sigma /dp\right)}_{T}<0$) results as a rule in a decrease of the surface tension. Such dependence can be interpreted as a particular realization of the principle of Le Chatelier–Braun [23] according to which the reaction of a system to external changes (increase of degree of metastability) causes a response (change of the interfacial energy favoring nucleation) counteracting it. This result is in line with the majority of theoretical and experimental investigations proving the decrease of the melting entropy with the size of a crystallite [68,69,70]. Note, however, that also alternative results have been reported [71], and they can be interpreted by specific features of the nanoclusters like clusters *“having geometries that are substantially different from the bulk material”*, as formulated as an explanation in the latter cited paper. In order to explain such effects in terms of Gibbs’ theory, we have, however, to go beyond Gibbs’ standard treatment employed in CNT as discussed in the Introduction to the present paper. As mentioned there, this is a topic beyond the scope of the present analysis. Experimental data on crystal nucleation rates and the dependence on temperature [57,72,73,74,75,76,77,78,79,80,81] supply us with an additional confirmation of our theoretical results, showing that the mentioned additional effects can be actually neglected in a variety of applications.

A detailed molecular dynamics (MD) analysis of the mechanism of crystal nucleation in one-component systems and the basic thermodynamic parameters affecting it was performed by one of us and colleagues in [63,82,83,84,85,86]. The respective analysis was devoted to MD simulations modeling of the kinetics of spontaneous crystal nucleation in under-cooled one-component Lennard–Jones liquids and a detailed comparison with the basic assumptions and results of CNT. In particular, the interfacial energy density of the critical crystal nucleus was determined. Simultaneously, the interfacial energy density was computed by MD methods for the planar liquid–crystal interface [85]. It was found that for typical sizes of the critical nuclei in the range of 0.7–1.0 nm, the value of the effective specific interfacial energy differed from that of the planar interface by less than 15%. A comparison of the MD-results with the classical nucleation theory shows that for the considered case of crystallization of one-component liquids, they are in good agreement not only with respect to the final result, the nucleation rate, but also with respect to the parameters entering it. Consequently, the results of MD simulations of crystallization in such simple one-component liquids demonstrate the validity of the basic assumptions and the final results of CNT for this particular case of phase formation. It is shown there that an increase of temperature leads to a monotonic increase of the interfacial free energy of the different orientations of the crystal phase at liquid–crystal equilibrium coexistence [85]. Similarly, the surface energy of the crystal-nucleus interface increases with increasing temperature. Both findings are in line with our analytical results. The effective surface energy of crystal-phase critical nuclei decreases linearly with increasing pressure in cases when pressure increase results in an increase of the degree of metastability. These results have been reconfirmed and further advanced in [87]. A linear increase of the surface tension with temperature was obtained by MD simulations of Ni and Al recently in [88]. A detailed comparison with estimates of the pressure and temperature dependence of the surface tension in application to ice-crystallization (see, e.g., [89,90,91,92]), being also in line with our results, will be presented in an accompanying paper [42]. Note that due to the specific properties of water, an increase of the degree of metastability is connected there with a decrease of pressure.

**Figure 1:**
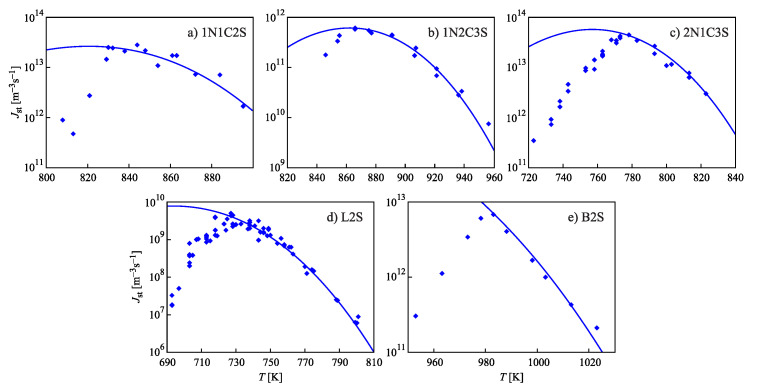
Steady-state nucleation rates of several glass-forming melts and their interpretation via Equation (71) utilizing the Tolman equation with parameters as given in Table 1 (see also the text and the caption to Table 1 for more details). As is evident, the Tolman equation with appropriate values of $\sigma_{\infty }$ and δ allows a good description of the experimental data down to temperatures corresponding to the maximum of the steady-state nucleation rate. For lower temperatures, additional factors affecting nucleation have to be accounted for going beyond CNT (for details, see [6,7,32,33,34]).

In [32,33,34], experimental data on the steady-state nucleation rate,

(71) $$J=J_{0}\exp\left(-\frac{W_{c}}{k_{B}T}\right)$$

for different glass-forming systems were interpreted in terms of CNT utilizing the Tolman equation, Equation (3), for the description of the curvature dependence of the surface tension. In this approach, $\sigma_{\infty }$ and δ were taken as fit parameters. The results are shown in Figure 1. It is evident that the Tolman equation allows us to interpret the experimental data with dependence on temperature with high accuracy down to values of temperature corresponding to the maximum of the steady-state nucleation rate.

On the other hand, we can now try to describe the experimental data by determining δ via Equation (69). This relation allows one to determine the combination $\left(d_{0}/\delta \right)\sigma_{\infty }$ via directly-measurable properties of both phases. The results are shown for several glass-forming melts in Table 1. As it turns out, the values of this combination obtained via the mentioned fit procedure and via Equation (69) are quite near each other. As a consequence, by utilizing Equation (69), we can describe the nucleation rate and related data in a first approximation with only one fit parameter, $\sigma_{\infty }$, but account via the mentioned approach also for the curvature dependence of the surface tension. Note that the theoretical estimates have been computed for real systems utilizing only directly-measurable parameters of the substances under consideration. In Table 1, the data are given for crystallization caused by variations of temperature. We expect a similar coincidence for crystallization caused by variations of pressure. A detailed analysis of the latter problems is considered as highly interesting.

However, although the values of the terms $\left(d_{0}\sigma_{\infty }/\delta \right)$ obtained from experiment and by theoretical computations in application to crystallization are quite near to each other, the resulting curves for the steady-state nucleation rate differ partly significantly from the fitting curves shown in Figure 1. By choosing an appropriate value of $\sigma_{\infty }$, one can bring the results to coincidence for the range of the maximum nucleation rates, but then, the results differ for higher values of temperature or vice versa. Consequently, in the further development of the theory outlined here, by utilizing appropriate generalizations of the Tolman equation as partly described here earlier, one can try to advance a more precise description. More details concerning this procedure can be found in [15] (Unfortunately, in the latter cited paper, the number one in one of the equations (Equation (69) here) was incorrectly omitted, and the respective procedure has to be repeated. This task is planned to be performed in a forthcoming study).

As already discussed in the Introduction and elaborated in more detail in Section 4, the situation with the curvature dependence of the surface tension is quite different in the description of condensation and boiling. Here, correction terms are absolutely essential in order to arrive at an at least qualitatively correct description of the curvature dependence of the surface tension in application to nucleation processes. This difference is caused by the existence of a spinodal and the resulting from it consequences for the dependence of the surface tension on the pressure or temperature for metastable states of the ambient fluid. Its dependence on cluster size for condensation and boiling can, as will be shown, appropriately be described by a generalization of Equations (4) or (5). Prior to that, we would like to draw attention to some problematic, from our point of view, statements concerning the Tolman equation and the Tolman parameter in crystal nucleation published quite recently.

### 3.4. Critical Analysis of Some Alternative Approaches

In [20], Gunawardana and Xueyu Song discussed the applicability of the Tolman equation to the description of crystal nucleation at fixed pressure and varying temperature. Their aim was formulated as follows: *“In this paper, a theoretical model is developed and tested to calculate the curvature dependence of γ at a crystal-liquid interface. The first order correction to the $\gamma_{0}$ due to the curvature (1/R, R is the radius of the crystallite) can be expressed as*

(72) $$\gamma \left(R,T\right)=\gamma_{0}\left(T\right)\left(1+\frac{2\delta }{R}\right)\;,$$

*where δ is called the Tolman length [11] in analogous to the liquid-vapor interface. To the lowest order, we assume that the temperature dependence of δ is negligible. As the main result of this paper, an analytical expression for the constant δ is derived from an equilibrium thermodynamic approach. Then the value of δ is calculated using a spherical shape crystalline cluster which coexists with its liquid in atomistic simulations”*. In our comments, we retain here their notations for the surface tension, γ, and its value for a planar equilibrium coexistence of liquid and crystal, $\gamma_{0}$, in order to compare their with our results using for that purposes the introduced earlier notations σ and $\sigma_{\infty }$.

The mentioned authors (i) did not clarify in their paper why the reduction of the description of critical crystallites to a simplified model with a radius *R* is possible at all. Only performing in advance such an analysis, all questions concerning the uniqueness of the definition of the parameter δ discussed by them in the final part of their paper could be excluded from the very beginning. Moreover, (ii) they did not derive one of their basic equations, here reproduced as Equation (72), but postulated it, referring to Tolman’s analysis. As noted here earlier, Tolman analyzed phase formation caused by the variation of pressure at constant temperature. For this reason, in their analysis, the questions remain open whether Tolman’s equation is applicable at all to crystallization initiated by variations of temperature and which parameters have actually to be determined. As shown by us, Tolman’s approach can be employed resulting for the process considered by the authors into the following relations (see Equations (3) and (51)):

(73) $$\sigma \left(R\right)=\frac{\sigma_{\infty }}{1+\frac{2\delta }{R}}\;,\;\sigma_{\infty }=\sigma_{\infty }\left(T_{eq},p_{eq}\right)\;,\;\delta =\delta_{\infty }\left(T_{eq},p_{eq}\right)\;,$$

(74) $$\delta_{\infty }\left(T_{eq},p_{eq}\right)=\lim_{R\to \infty }\frac{{\tilde{v}}_{\alpha }\left[\left(S_{\sigma }/A\right)-\left(n_{\sigma }/A\right){\tilde{s}}_{\beta }\right]}{{\tilde{s}}_{\alpha }-{\tilde{s}}_{\beta }}\;.$$

The authors refer to Tolman’s original paper (here, [11]) and set Γ=0 anyway. Such an approach is highly questionable since in such a case, in Tolman’s work, δ(R) is equal to zero (see Equations (2) and (47)). At least, in the correct description, Equation (74), one substantial contribution is omitted. (iii) According to Equations (52) and (53), at constant temperature, the surface tension is a function either of pressure or of radius, allowing Tolman to formulate his dependence σ=σ(R). Similarly, at constant pressure, the surface tension is either a function of radius or temperature, but not of both parameters, as suggested by the authors in their equation, here given as Equation (72). Moreover, (iv) $\sigma_{\infty }$ (or $\gamma_{0}$) has (at constant pressure) one and only one value and cannot be treated as a function of temperature. Treating the dependence of the surface tension on radius, as stated by the authors, in the lowest order of 1/R, then (v) δ is by definition of the Tolman parameter independent of temperature, and this does not have to be proposed artificially. (vi) This limiting value of the parameter $\delta =\delta_{\infty }\left(T_{eq},p_{eq}\right)$ has to be computed for a planar equilibrium coexistence of liquid and crystal and not, as done by the authors, for a crystallite of finite size in the liquid. Provided the values of δ, given by Equation (47) or (51), could be computed for all values of cluster size in the interval of cluster size, (R,∞), these values cannot be employed for the specification of the parameter δ in Equation (3) or (5). Instead, it has to be utilized to solve the differential equation, Equation (1). Its solution would lead, in general, to results quite different from the Tolman equation (see, e.g., [12,13]). Of course, in an alternative approach as discussed by us, the Tolman equation can be generalized as expressed by Equations (4) and (5). However, in such a case, (vii) the generalized Tolman parameter is a function of the temperature or cluster radius and cannot be constant. Finally, the original form of the Tolman equation is Equation (73). Utilizing instead the approximation Equation (72), (viii) the Tolman parameter δ employed by the authors is actually the Tolman parameter multiplied by minus one.

In their analysis, Gunawardana and Xueyu Song tried to formulate a differential equation for the change of the surface tension with temperature or critical cluster size, computing the derivative of γ in Equation (72) with respect to temperature. They obtained (assuming δ to be independent of and $\gamma_{0}$ to be dependent on temperature):

(75) $$\frac{d\gamma }{dT}=\frac{d\gamma_{0}}{dT}\left(1+\frac{2\delta }{R}\right)-\frac{2\gamma_{0}\delta }{R^{2}}\frac{dR}{dT}\;.$$

The change of the surface area and, consequently, of the radius with temperature they estimated from the respective density variations of the bulk solid along the coexistence path (Equation (23) in [20]). Even provided Equation (75) would be true, (ix) such an approach is not correct. *R* is the critical cluster radius and has to be determined correctly via the equilibrium conditions. In general, the variation of the radius of the critical crystallite may be caused by both variations of the density, but also by changes of the number of particles in the critical clusters. Moreover, how can one seriously assume in the derivation the parameter δ to be independent of temperature, obtaining as a consequence of such an assumption a result (Equation (22) in [20]) where δ depends significantly on temperature? However, (x) even more serious problems exist with the herein discussed approach. Generally, it is always highly questionable to try to establish the differential equation describing a certain dependence having at one’s disposal only a very approximate solution. Moreover, as discussed in connection with Equations (73) and (74), $\sigma_{\infty }=\sigma_{\infty }\left(T_{eq},p_{eq}\right)$ and (since $\gamma_{0}$ is in their notation identical to $\sigma_{\infty }\left(T_{eq},p_{eq}\right)$ as denoted by us) in Equation (75), the authors had to set $(d\gamma_{0}/dT)=0$. The solution of Equation (75) corrected in such way is quite different from Equation (72) with which the authors started. Finally, there is no need to try to establish in an artificial way the differential equation for the description of the curvature dependence of the surface tension. Employing Gibbs’ theory, for crystallization caused by the variation of temperature, it is given by an equation formulated already by Gibbs and then by Tolman, Equation (1), however as shown here with a value of δ given by Equation (51). Consequently, the approach followed by the authors and their main result (as formulated by them) were incorrect.

Some other problematical aspects of the paper also have to be noted: (xi) The Tolman parameter in both its traditional definition as formulated by Tolman (Equation (3) or, in the application considered here, Equations (73) and (74)) is a function of pressure and temperature for the respective states of equilibrium coexistence of liquid and crystal. It is a function of temperature or cluster size in the generalization given by Equation (5). The statement in the abstract *“The correction parameter (δ), which is analogous to the Tolman length at a liquid-vapor interface, is found to be 0.48 ± 0.05 for a Lennard-Jones (LJ) fluid”* should have been supplemented by the clarification “for the only one considered by us temperature”. The assumption followed by them in the analysis that it always has the same value is not correct. (xii) Assuming that for their simulation with 99,159 particles, macroscopic equilibrium is reached (Figure 1 in [20]), the surface tension for the equilibrium coexistence of crystal and liquid at a planar interface is estimated in reduced Lennard–Jones units as $\gamma_{0}=0.368$ at $T_{0}=0.64$. This is the value one has to use then in the expression for the work of critical cluster formation and not its value at a temperature below $T_{0}$, i.e., taking $\gamma_{0}=0.340$ at the temperature T=0.58. This incorrect approach they had to compensate by curvature corrections to the surface tension, resulting in an increase of the surface tension with decreasing temperature or decreasing critical cluster size in order to arrive at an agreement between theory and simulation. This result is in contradiction with experimental data, molecular dynamics simulations, and theoretical predictions as summarized above and reconfirmed quite recently in [80,87]. (xii) Finally, being able to describe nucleation rate data by reaching an agreement between theory and experiment for only one selected temperature is commonly not considered as a convincing proof of the validity of the theoretical approach. Some more critical comments on the paper by Gunawardana and Xueyu Song [20] were given in [100].

In [21], B. Cheng and M. Ceriotti advanced an alternative approach to the determination of the Tolman length. They motivated their analysis by the importance of curvature corrections to the understanding of nucleation and coarsening. While the first statement is true, (i) in Ostwald ripening, curvature corrections to the surface tension do not play any significant role due to the relatively large average size of the clusters [101]. Further, they stated that in addition to the surface tension, the equimolecular dividing surface was introduced by Gibbs. The authors specified it by the condition that no surface excess of volume had to be accounted for if this particular dividing surface was chosen. However, (ii) for any dividing surface, the interface has a zero volume in the Gibbs approach. Moreover, they noted that the equimolecular dividing surface is commonly used when analyzing nucleation. This statement is also not correct. (iii) Almost all attempts to interpret nucleation data in terms of CNT utilize the surface of tension. In particular, the Gibbs–Tolman approach to the description of the curvature dependence of the surface tension also employs the surface of tension as the dividing surface and not the equimolecular dividing surface. With reference to Tolman [11], the authors stated that, for planar interfaces, the Tolman length is the difference between the location of the surface of tension and the equimolecular dividing surface. Again, (iv) this is true only for cases when the supersaturation is varied by changes of external pressure and not generally (see Equation (74)). It does not hold for phase formation caused by variations of temperature studied by the authors (see their Figure 1 in [21]).

Similar problems can be found also in the determination of the Tolman parameter in application to condensation and boiling. For example, in [22], the authors estimated the liquid–vapor surface tension from simulations of TIP4P/2005 water nano-droplets of sizes N=100–2880 molecules over a temperature range T≅180–300 K. In their “thermodynamic route” of computations, they assumed δ to be determined via $\delta =(R_{e}-R_{s})$ (their Equation (3)). The equimolecular dividing surface was not correctly defined, but this was not the main problem; the main problem was that for the case of phase formation they considered (change of the degree of metastability by variation of temperature), δ is not given by their Equation (3), but by Equation (51) of the present paper. The way how our method can be applied to condensation and boiling processes will be addressed in the next section.

## 4. Application to Condensation and Boiling

### 4.1. Bulk Properties of Ambient and Newly-Evolving Phases, Binodal and Spinodal Curves

The correlation between capillarity and the entropy of the phase transformation has been so far widely discussed preferentially in application to crystal nucleation. This interest was caused by the absence of experimental data for the surface tension governing crystal nucleation. For this reason, estimates of the value of the surface tension for the equilibrium coexistence of liquid and crystal at planar interfaces have been advanced via the Stefan–Skapski–Turnbull relation, Equations (28) or (29). In the present paper, we would like to analyze to what extent it can be of use also for the description of critical cluster formation in condensation and boiling.

As an example, we consider here one-component fluids described by the van der Waals equation of state. In dimensionless variables, this equation has the form [23,102]:

(76) $$\Pi \left(\omega ,\theta \right)=\frac{8\theta }{3\omega -1}-\frac{3}{\omega^{2}}\;,$$

(77) $$\Pi \equiv \frac{p}{p_{c}}\;\;\omega \equiv \frac{\tilde{v}}{{\tilde{v}}_{c}}\;,\;\theta \equiv \frac{T}{T_{c}}\;,$$

where $\tilde{v}$, *p*, and *T* are the volume per particle, pressure, and temperature and by ${\tilde{v}}_{c}$, $p_{c}$, and $T_{c}$, the values of the same parameters in the critical point are denoted. As far as required in the computations, we will assign to the critical parameters the values $T_{c}=647.1$ K, $p_{c}=22.064\cdot 10^{6}$ N/m${}^{2}$, and ${\tilde{v}}_{c}=9.3\cdot 10^{-29}$ m${}^{3}$, referring to water.

The chemical potential of the van der Waals fluid can be written as [103,104]:

(78) $$\frac{\mu (\theta ,\omega )}{p_{c}{\tilde{v}}_{c}}=-\frac{8\theta }{3}\ln\left(3\omega -1\right)+\frac{8\theta \omega }{3\omega -1}-\frac{6}{\omega }+\chi \left(\theta \right)\;.$$

Here, χ(θ) is some function of temperature, its form is irrelevant for the subsequent analysis. By reformulating Equation (16) in reduced variables defined via Equation (77), we obtain:

(79) $$d\left(\frac{\mu (\theta ,\Pi )}{p_{c}{\tilde{v}}_{c}}\right)=-\left(\frac{T_{c}}{p_{c}{\tilde{v}}_{c}}\right)\tilde{s}d\theta +\omega d\Pi \;,$$

(80) $$\tilde{s}=-\left(\frac{p_{c}{\tilde{v}}_{c}}{T_{c}}\right){\left.\frac{\partial }{\partial \theta }\left(\frac{\mu (\theta ,\Pi )}{p_{c}{\tilde{v}}_{c}}\right)\right|}_{\Pi }\;.$$

In order to obtain expressions for the entropy per particle of a van der Waals fluid, we have to express ω in Equation (78) as a function of (θ,Π) as given by Equation (76) and then perform the respective computations. Accounting in Equation (78) for the dependence μ=μ(θ,ω(θ,Π)), we may write directly:

(81) $${\left.\frac{\partial \tilde{\mu }\left(\theta ,\omega \left(\theta ,\Pi \right)\right)}{\partial \theta }\right|}_{\Pi }={\left.\frac{\partial \tilde{\mu }}{\partial \theta }\right|}_{\omega }+{\left.\frac{\partial \tilde{\mu }}{\partial \omega }\right|}_{\theta }{\left.\frac{\partial \omega }{\partial \theta }\right|}_{\Pi }\;,\;\tilde{\mu }=\frac{\mu (\theta ,\omega )}{p_{c}{\tilde{v}}_{c}}\;.$$

A combination of Equations (76) and (78) yields:

(82) $$\frac{\mu (\theta ,\omega )}{p_{c}{\tilde{v}}_{c}}=-\frac{8\theta }{3}\ln\left(3\omega -1\right)+\omega \Pi -\frac{3}{\omega }+\chi \left(\theta \right)\;.$$

Equations (80)–(82) result in:

(83) $$\tilde{s}=-\left(\frac{p_{c}{\tilde{v}}_{c}}{T_{c}}\right)\left\{\left[-\frac{8}{3}\ln\left(3\omega -1\right)+\frac{d\chi (\theta )}{d\theta }\right]+\left[-\frac{8\theta }{3\omega -1}+\Pi +\frac{3}{\omega^{2}}\right]{\left.\frac{\partial \omega }{\partial \theta }\right|}_{\Pi }\right\}\;.$$

Accounting for Equation (76), we finally obtain:

(84) $$\tilde{s}=-\left(\frac{p_{c}{\tilde{v}}_{c}}{T_{c}}\right)\left\{-\frac{8}{3}\ln\left(3\omega -1\right)+\frac{d\chi (\theta )}{d\theta }\right\}\;.$$

Utilizing the same procedure, we can then obtain the expression for the specific heat per particle of a van der Waals fluid via:

(85) $${\tilde{c}}_{p}=T{\left(\frac{\partial \tilde{s}}{\partial T}\right)}_{p}=\theta {\left(\frac{\partial \tilde{s}}{\partial \theta }\right)}_{\Pi }=\theta \left({\left.\frac{\partial \tilde{s}}{\partial \theta }\right|}_{\omega }+{\left.\frac{\partial \tilde{s}}{\partial \omega }\right|}_{\theta }{\left.\frac{\partial \omega }{\partial \theta }\right|}_{\Pi }\right)\;.$$

Employing Equation (84), Equation (85) leads to:

(86) $${\tilde{c}}_{p}=\theta \left(\frac{p_{c}{\tilde{v}}_{c}}{T_{c}}\right)\left(\frac{8}{3\omega -1}{\left.\frac{\partial \omega }{\partial \theta }\right|}_{\Pi }-\frac{d^{2}\chi \left(\theta \right)}{d\theta^{2}}\right)$$

with:

(87) $${\left.\frac{\partial \omega }{\partial \theta }\right|}_{\Pi }=\frac{4\omega^{3}\left(3\omega -1\right)}{3\left[4\theta \omega^{3}-{\left(3\omega -1\right)}^{2}\right]}\;.$$

Finally, we obtain:

(88) $${\tilde{c}}_{p}=\left(\frac{p_{c}{\tilde{v}}_{c}}{T_{c}}\right)\left(\frac{32\theta \omega^{3}}{3\left[4\theta \omega^{3}-{\left(3\omega -1\right)}^{2}\right]}-\theta \frac{d^{2}\chi \left(\theta \right)}{d\theta^{2}}\right)\;.$$

The position of the spinodal, the border between the thermodynamically-metastable and -unstable states in the absence of heterogeneous nucleation centers, is given by the equation:

(89) $$\frac{d}{d\omega }\Pi \left(\theta ,\omega \right)=0\;.$$

For any value of temperature below the critical temperature ($\theta <\theta_{c}=1$), Equation (89) yields two solutions, which coincide at the critical point. The location of the binodal curve is determined by the conditions of thermodynamic equilibrium of vapor (gas) and liquid at a planar interface (equality of pressure and chemical potential), that is by the solution of the system of equations:

(90) $$\Pi \left(\theta ,\omega_{gas}\right)=\Pi \left(\theta ,\omega_{liq}\right)\;,\;\mu \left(\theta ,\omega_{gas}\right)=\mu \left(\theta ,\omega_{liq}\right)\;.$$

**Figure 2:**
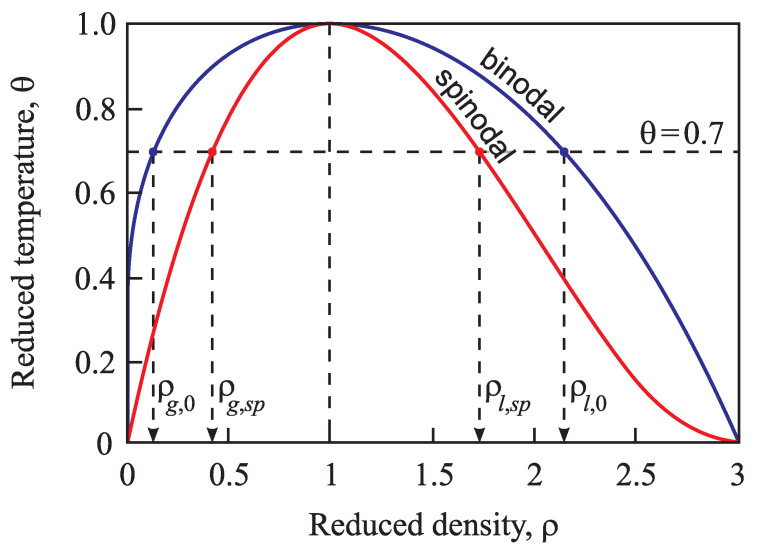
Location of the binodal and spinodal curves for a van der Waals fluid (see also the text).

Similar to the above-discussed case, for any value of temperature in the range $0<\theta <\theta_{c}=1$, Equation (90) yields one solution for $\omega_{g}$ and one for $\omega_{l}$. These two solutions coincide at the critical point, again. The binodal and spinodal curves are given in terms of reduced density, ρ=1/ω, in Figure 2.

Numerical computations are frequently performed here assuming the reduced temperature to be equal to θ=0.7. In such a case, the values of the reduced volume at the binodal ($\omega_{b}$) and spinodal ($\omega_{sp}$) curves are given by:

(91) $$\omega_{b}^{\left(left\right)}=0.467\;,\;\omega_{b}^{\left(right\right)}=7.811\;,$$

(92) $$\omega_{sp}^{\left(left\right)}=0.579\;,\;\omega_{sp}^{\left(right\right)}=2.376\;.$$

Accordingly, the reduced equilibrium densities, ρ=1/ω, of liquid ($\rho_{l,0}$) and vapor ($\rho_{g,0}$) are:

(93) $$\rho_{l,0}={\left(\omega_{b}^{\left(left\right)}\right)}^{-1}=2.14\;,\;\rho_{g,0}={\left(\omega_{b}^{\left(right\right)}\right)}^{-1}=0.128\;,$$

and the densities at the liquid ($\rho_{l,sp}$) and vapor branches ($\rho_{g,sp}$) of the spinodal curve are:

(94) $$\rho_{l,sp}={\left(\omega_{sp}^{\left(left\right)}\right)}^{-1}=1.727\;,\;\rho_{g,sp}={\left(\omega_{sp}^{\left(right\right)}\right)}^{-1}=0.421\;.$$

The non-equilibrium values of density of liquid and vapor will be denoted as $\rho_{l}$ and $\rho_{g}$, respectively. An illustration of these notations and results is given in Figure 2.

### 4.2. Surface Tension

#### 4.2.1. Dependence of the Surface Tension on Pressure and Temperature

The Tolman parameter δ we determine here first in its original meaning for the limit of large critical cluster sizes ($\delta \left(R\to \infty \right)=\delta_{\infty }$) considering small deviations from thermodynamic equilibrium specified by the temperature, $T_{b}$, and the pressure, $p_{b}$, corresponding to states along the binodal curve. In such cases, the basic assumptions of CNT are fulfilled [3,4,103,104,105], the intensive state parameters of the critical clusters are widely identical to the state parameters of the newly-evolving macroscopic phase, and the parameters of the critical clusters are obtained by the equilibrium conditions as advanced by Gibbs in his classical treatment of interfacial phenomena [1].

For the determination of the dependence of the surface tension of droplets and bubbles of critical size on pressure and temperature, we employ here the approach developed by us in the application to crystal nucleation in [24,25,26] and advanced here. We arrive, again, at:

(95) $$\frac{\sigma (T,p)}{\sigma_{\infty }\left(T_{b},p_{b}\right)}≅\frac{T\left({\tilde{s}}_{\beta }\left(T,p\right)-{\tilde{s}}_{\alpha }\left(T,p\right)\right)}{T_{b}\left({\tilde{s}}_{\beta }\left(T_{b},p_{b}\right)-{\tilde{s}}_{\alpha }\left(T_{b},p_{b}\right)\right)}\;,$$

and, similar to Equation (35), at:

(96) $$\frac{\sigma (T,p)}{\sigma (T_{b},p_{b})}≅\frac{T}{T_{b}}\left(1-\frac{\Delta {\tilde{c}}_{p}\left(T_{b},p_{b}\right)}{\Delta \tilde{s}\left(T_{b},p_{b}\right)}\frac{T_{b}-T}{T_{b}}-\frac{p_{b}\Delta \left[\tilde{v}\left(T_{b},p_{b}\right){\tilde{\alpha }}_{p}\left(T_{b},p_{b}\right)\right]}{\Delta \tilde{s}\left(T_{b},p_{b}\right)}\left(\frac{p-p_{b}}{p_{b}}\right)\right)$$

with:

$$\Delta \tilde{s}\left(T,p\right)={\tilde{s}}_{\beta }\left(T,p\right)-{\tilde{s}}_{\alpha }\left(T,p\right)\;,$$

(97) $${\tilde{c}}_{p}\left(T,p\right)=T\left(\frac{\partial \tilde{s}}{\partial T}\right)\;,\;\Delta {\tilde{c}}_{p}\left(T,p\right)={\tilde{c}}_{p\beta }\left(T,p\right)-{\tilde{c}}_{p\alpha }\left(T,p\right)\;,$$

$${\tilde{\alpha }}_{p}\left(T,p\right)=\frac{1}{\tilde{v}}{\left(\frac{\partial \tilde{v}}{\partial T}\right)}_{p}\;,\;\Delta \left[\tilde{v}\left(T,p\right){\tilde{\alpha }}_{p}\left(T,p\right)\right]={\tilde{v}}_{\beta }\left(T,p\right){\tilde{\alpha }}_{p\beta }\left(T,p\right)-{\tilde{v}}_{\alpha }\left(T,p\right){\tilde{\alpha }}_{p\alpha }\left(T,p\right)\;.$$

**Figure 3:**
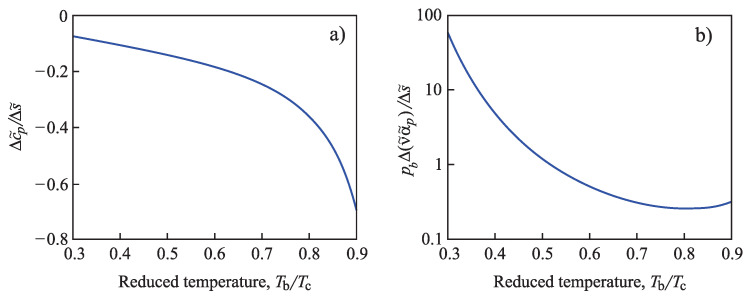
Parameters $\left(\Delta {\tilde{c}}_{p}\left(T_{b},p_{b}\right)/\Delta \tilde{s}\left(T_{b},p_{b}\right)\right)$ (**a**) and $\left(p_{b}\Delta \left(\tilde{v}{\tilde{\alpha }}_{p}\left(T_{b},p_{b}\right)\right)/\Delta \tilde{s}\left(T_{b},p_{b}\right)\right)$ (**b**) with dependence on the reduced temperature $\theta =\theta_{b}$. For any value of $\theta =\theta_{b}$, the pressure $p_{b}$ is computed via Equation (90), determining the binodal curve of the van der Waals fluid.

The ratios of the coefficients $\left(\Delta {\tilde{c}}_{p}\left(T_{b},p_{b}\right)/\Delta \tilde{s}\left(T_{b},p_{b}\right)\right)$ and $\left(p_{b}\Delta \left(\tilde{v}\left(T_{b},p_{b}\right){\tilde{\alpha }}_{p}\left(T_{b},p_{b}\right)\right)/\Delta \tilde{s}\left(T_{b},p_{b}\right)\right)$ in Equation (96) can be determined employing Equations (76), (84) and (88). The results are shown for different values of the reduced temperature $\theta =\theta_{b}$ in Figure 3. For any value of $\theta =\theta_{b}$, the pressure $p_{b}$ is computed via Equation (90), determining the binodal curve of the van der Waals fluid.

**Figure 4:**
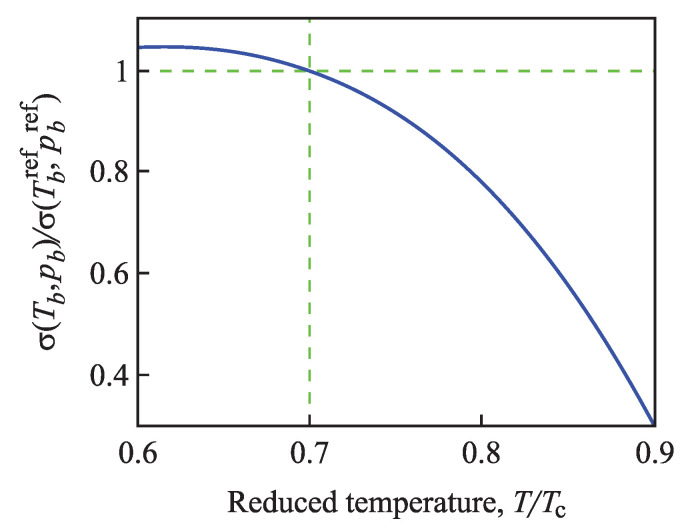
Change of the surface tension along the binodal curve computed via Equation (96). As the reference state, the temperature $T_{b}^{\mathrm{ref}}=0.7T_{c}$ and the corresponding value of pressure, $p_{b}^{\mathrm{ref}}$, along the binodal curve were chosen.

Having at one’s disposal these ratios, we can determine via Equation (96) the surface tension of critical clusters in dependence on pressure and temperature. In particular, we can also predict how the surface tension is changed along the binodal curve. For such purposes, we select a particular reference state, $\left(T_{b}^{\mathrm{ref}},p_{b}^{\mathrm{ref}}\right)$, along the binodal curve and determine then the ratio of the values of the surface tension for different values of temperature and pressure along the binodal. For an illustration, in Figure 4, we take as the reference state $T=0.7T_{c}$ and the respective value of pressure and compute the ratio $\sigma \left(T_{b},p_{b}\right)/\sigma_{\mathrm{ref}}$ in dependence on temperature and pressure along the binodal curve.

#### 4.2.2. Determination of the Tolman Parameter

In order to determine the Tolman parameter, $\delta =\delta_{\infty }=\delta \left(R\to \infty \right)$, in Equation (3), we rewrite this equation in the form:

(98) $$\delta_{\infty }\left(T_{b},p_{b}\right)=\lim_{R\to \infty }\left\{\frac{R}{2}\left(\frac{\sigma_{\infty }\left(T_{b},p_{b}\right)}{\sigma (R)}-1\right)\right\}=\lim_{R\to \infty }\left\{\frac{R}{2\sigma }\sigma_{\infty }\left(1-\frac{\sigma }{\sigma_{\infty }\left(T_{b},p_{b}\right)}\right)\right\}\;.$$

In addition to Equation (96), we employ the equilibrium conditions:

(99) $$p_{\alpha }-p=\frac{2\sigma }{R}\;,\;\mu_{\alpha }\left(T,p_{\alpha }\right)=\mu_{\beta }\left(T,p\right)\;.$$

**Figure 5:**
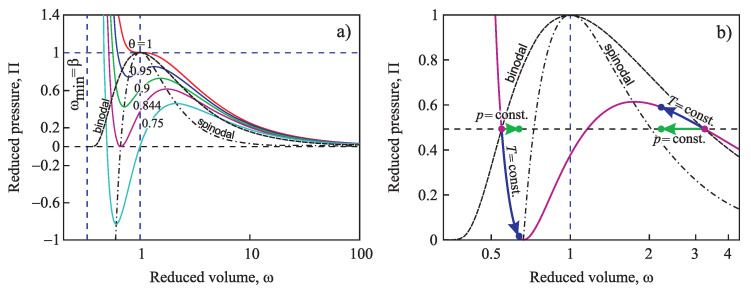
Four possible paths (two for boiling and two for condensation) to generate metastable initial states caused by either variation of pressure at constant temperature or variation of temperature at constant pressure analyzed in the present paper. (**a**) Different isotherms of the van der Waals fluid, (**b**) Illustration of the different ways to generate metastable states.

Let us consider first condensation and boiling at $T=T_{b}$ caused by variation of pressure, i.e., moving along the respective isotherm (see Figure 5). In such a case, we may replace the pressure difference via:

(100) $$\mu_{\alpha }\left(T_{b},p_{\alpha }\right)=\mu_{\alpha }\left(T_{b},p_{b}\right)+{\tilde{v}}_{\alpha }\left(T_{b},p_{b}\right)\left(p_{\alpha }-p_{b}\right)\;,$$

(101) $$\mu_{\beta }\left(T_{b},p\right)=\mu_{\beta }\left(T_{b},p_{b}\right)+{\tilde{v}}_{\beta }\left(T_{b},p_{b}\right)\left(p-p_{b}\right)\;,$$

resulting in:

(102) $${\tilde{v}}_{\alpha }\left(T_{b},p_{b}\right)\left(p_{\alpha }-p_{b}\right)={\tilde{v}}_{\beta }\left(T_{b},p_{b}\right)\left(p-p_{b}\right)\;.$$

or:

(103) $$p_{\alpha }-p=\left(p_{b}-p\right)\left(1-\frac{{\tilde{v}}_{\beta }\left(T_{b},p_{b}\right)}{{\tilde{v}}_{\alpha }\left(T_{b},p_{b}\right)}\right)\;.$$

Equations (96), (98) and (99) yield:

(104) $$\delta_{\infty }^{\left(p\right)}\left(T_{b},p_{b}\right)=\frac{\sigma_{\infty }\left(T_{b},p_{b}\right){\tilde{v}}_{\alpha }\left(T_{b},p_{b}\right)}{p_{b}\left[{\tilde{v}}_{\beta }\left(T_{b},p_{b}\right)-{\tilde{v}}_{\alpha }\left(T_{b},p_{b}\right)\right]}\left(\frac{p_{b}\Delta \left[\tilde{v}\left(T_{b},p_{b}\right){\tilde{\alpha }}_{p}\left(T_{b},p_{b}\right)\right]}{\Delta \tilde{s}\left(T_{b},p_{b}\right)}\right)\;.$$

Similarly, we may proceed considering condensation and boiling at $p=p_{b}$ caused by variations of temperature (see, again, Figure 5 for an illustration). Here, we may write:

(105) $$\mu_{\alpha }\left(T,p_{\alpha }\right)=\mu_{\alpha }\left(T_{b},p_{b}\right)+{\tilde{v}}_{\alpha }\left(T_{b},p_{b}\right)\left(p_{\alpha }-p_{b}\right)-{\tilde{s}}_{\alpha }\left(T_{b},p_{b}\right)\left(T-T_{b}\right)\;,$$

(106) $$\mu_{\beta }\left(T,p_{b}\right)=\mu_{\beta }\left(T_{b},p_{b}\right)-{\tilde{s}}_{\beta }\left(T_{b},p_{b}\right)\left(T-T_{b}\right)\;,$$

resulting in

(107) $$p_{\alpha }-p_{b}=-\frac{{\tilde{s}}_{\beta }\left(T_{b},p_{b}\right)-{\tilde{s}}_{\alpha }\left(T_{b},p_{b}\right)}{{\tilde{v}}_{\alpha }\left(T_{b},p_{b}\right)}\left(T-T_{b}\right)\;.$$

Equations (96), (98) and (99) yield in the limit $T\to T_{b}$:

(108) $$\delta_{\infty }^{\left(T\right)}\left(T_{b},p_{b}\right)=\frac{\sigma_{\infty }\left(T_{b},p_{b}\right){\tilde{v}}_{\alpha }\left(T_{b},p_{b}\right)}{T_{b}\left({\tilde{s}}_{\beta }\left(T_{b},p_{b}\right)-{\tilde{s}}_{\alpha }\left(T_{b},p_{b}\right)\right)}\left(1+\frac{\Delta {\tilde{c}}_{p}\left(T_{b},p_{b}\right)}{\Delta \tilde{s}\left(T_{b},p_{b}\right)}\right)\;.$$

The differences $\Delta {\tilde{c}}_{p}$, $\Delta \tilde{s}$, and $\Delta (\tilde{v}{\tilde{\alpha }}_{p})$ have to be taken at a pressure and a temperature corresponding to the respective equilibrium state ($T_{b},p_{b}$).

**Figure 6:**
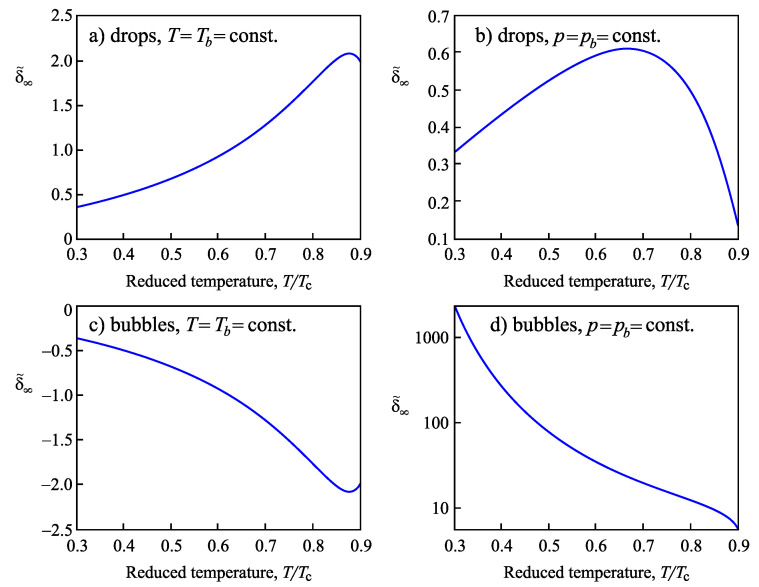
Change of the Tolman parameters in dependence on temperature and pressure along the binodal curve. As the reference state, the temperature $T_{b}^{\mathrm{ref}}=0.7T_{c}$ and the corresponding value of pressure $p_{b}^{\mathrm{ref}}$ along the binodal curve has been chosen. The value of the surface tension at this reference state is taken equal to $\sigma_{\mathrm{ref}}=0.1\;J/m^{2}$. The Tolman parameter is expressed in dimensionless units, ${\tilde{\delta }}_{\infty }\left(T_{b},p_{b}\right)=\delta_{\infty }\left(T_{b},p_{b}\right)/\sqrt[{3}]{{\tilde{v}}_{l}\left(T_{b}^{\mathrm{ref}},p_{b}^{\mathrm{ref}}\right)}$.

Following Stefan’s considerations (see Section 2.2) and the implementation as given here, we obtain for the Tolman parameter in application to condensation:

(109) $$\delta_{\infty }^{\left(p\right)}\left(T_{b},p_{b}\right)=\frac{\sigma_{\infty }\left(T_{b},p_{b}\right){\tilde{v}}_{l}\left(T_{b},p_{b}\right)}{p_{b}\left[{\tilde{v}}_{v}\left(T_{b},p_{b}\right)-{\tilde{v}}_{l}\left(T_{b},p_{b}\right)\right]}\left(\frac{p_{b}\Delta \left(\tilde{v}{\tilde{\alpha }}_{p}\right)}{\Delta \tilde{s}\left(T_{b},p_{b}\right)}\right)\;\mathrm{for}\;\mathrm{droplets}\;,$$

(110) $$\delta_{\infty }^{\left(T\right)}\left(T_{b},p_{b}\right)=\frac{\sigma_{\infty }\left(T_{b},p_{b}\right){\tilde{v}}_{l}\left(T_{b},p_{b}\right)}{T_{b}\left[{\tilde{s}}_{v}\left(T_{b},p_{b}\right)-{\tilde{s}}_{l}\left(T_{b},p_{b}\right)\right]}\left(1+\frac{\Delta {\tilde{c}}_{p}\left(T_{b},p_{b}\right)}{\Delta \tilde{s}\left(T_{b},p_{b}\right)}\right)$$

for both considered cases. As evident from Equations (109) and (110), the Tolman parameters are determined both via the bulk properties of the fluid under consideration and the value of the surface tension, all of them taken for pressure and temperature along the binodal curve. Their dependence on temperature and pressure along the binodal curve is illustrated in Figure 6. In the respective computations, the dependence of surface tension on temperature and pressure along the binodal curve is computed via Equation (96), again (see also Figure 4).

According to Gibbs’ theory of surface phenomena, the Tolman parameters for the description of the formation of critical droplets and bubbles are given by Equations (47) and (51). Equation (47) immediately yields $\delta_{\infty }^{\left(p\right)}\left(\mathrm{bubbles}\right)=-\delta_{\infty }^{\left(p\right)}\left(\mathrm{droplets}\right)$ or:

(111) $$\delta_{\infty }^{\left(p\right)}\left(T_{b},p_{b}\right)=-\frac{\sigma_{\infty }\left(T_{b},p_{b}\right){\tilde{v}}_{l}\left(T_{b},p_{b}\right)}{p_{b}\left[{\tilde{v}}_{v}\left(T_{b},p_{b}\right)-{\tilde{v}}_{l}\left(T_{b},p_{b}\right)\right]}\left(\frac{p_{b}\Delta \left(\tilde{v}{\tilde{\alpha }}_{p}\right)}{\Delta \tilde{s}\left(T_{b},p_{b}\right)}\right)\;\mathrm{for}\;\mathrm{bubbles}\;.$$

Setting in Equation (51) $S_{\sigma }≅n_{\sigma }{\tilde{s}}_{\alpha }$ (the superficial entropy per particle is assumed to be equal to the respective value of the cluster phase), we obtain with Equation (47) in the application to bubble formation:

(112) $$\delta_{\infty }^{\left(T\right)}≅\delta_{\infty }^{\left(p\right)}\left(1-\frac{{\tilde{v}}_{v}}{{\tilde{v}}_{l}}\right)\;,$$

(113) $$\delta_{\infty }^{\left(T\right)}\left(T_{b},p_{b}\right)=\sigma_{\infty }\left(T_{b},p_{b}\right)\left(\frac{\Delta (\tilde{v}{\tilde{\alpha }}_{p})}{\Delta \tilde{s}\left(T_{b},p_{b}\right)}\right)\;\mathrm{for}\;\mathrm{bubbles}\;.$$

The dependencies of the Tolman parameter on temperature for the case of the formation of bubbles are also shown in Figure 6. For the phase formation caused by the variation of pressure, the absolute value of the Tolman parameter is identical for condensation and boiling, in the alternative case, in general not. We will return to a discussion of this topic later.

#### 4.2.3. Generalization of the Tolman Equation in the Application to Condensation and Boiling: Account of the Existence of the Spinodal Curve

Utilizing Gibbs’ method of the description of surface phenomena [1], it can be shown that the surface tension (referring to the surface tension) approaches zero at the spinodal as:

(114) σ(R)→0,R→0,σ∝R,

i.e., the surface tension approaches zero linearly with the radius of the critical bubble, respectively the critical drop. This result was obtained first by Rusanov [46] using his modification of Gibbs’ theory. This conclusion is essentially based on the following argumentation: (i) For any state ($T_{sp},p_{sp}$) of the ambient fluid approaching the spinodal curve, the work of critical cluster formation, W=(1/3)σA, $A=4\pi R^{2}$ (Equation (7)), approaches zero [1]. For this reason, either the surface tension, or the radius of the critical cluster, or both have to approach zero. (ii) Utilizing Gibbs’ method for the determination of the properties of the critical clusters (drops or bubbles), even for such particular states along the spinodal curve, the thermodynamic driving force, ($p_{\alpha }-p_{sp}$) (see e.g., Equations (8), (9) and (99)), remains finite. (iii) As the only consequence, it follows that both the surface tension and the radius of the critical cluster have to approach zero in approaching the spinodal, but their ratio has to be finite, as expressed by Equation (114).

The type of behavior as described by Equation (114) is reflected correctly by the original version of the Tolman equation, Equation (3), but not by its extension, Equation (4). On the other hand, Equation (3) supplies us with the description of the curvature dependence of the surface tension in the limit R→∞ and not in the limit R→0. For this reason, another approximation for the curvature dependence of the surface tension in condensation and boiling is required if one wants to include in the description both limiting cases of behavior near a planar interface and in the approach to the spinodal curve. Such an equation can be derived based on the results outlined in [12,13].

In these papers [12,13], it was shown that a variety of equations proposed for the description of the curvature dependence of the surface tension can be described by the following ansatz for the function δ=δ(R) in Equations (1) and (2):

(115) $$\delta \left(R\right)=\delta_{\infty }\left(\frac{R+b}{R+a}\right)\;,$$

where *a* and *b* are parameters reflecting specific properties of the fluid under consideration. Such an ansatz results in dependencies σ=σ(R), which can be approximated by the relation:

(116) $$\sigma \left(R\right)=\frac{\sigma_{\infty }}{\sqrt{1+\frac{4\delta_{\infty }}{R}+{\left(\frac{L_{\infty }}{R}\right)}^{2}}}\;.$$

For the limiting cases, R→∞ and R→0, this relation yields:

(117) $$\sigma \left(R\right)≅\sigma_{\infty }\left(1-\frac{2\delta_{\infty }}{R}\right)\;\mathrm{for}\;R\to \infty \;,$$

(118) $$\sigma \left(R\right)≅\left(\frac{\sigma_{\infty }}{L_{\infty }}\right)R\;\mathrm{for}\;R\to 0\;,$$

in line with Equations (3) and (114). Utilizing the equilibrium conditions, Equation (99), we obtain the following estimate for the values of the parameter $L_{\infty }\left(p_{sp},T_{sp}\right)$ along the spinodal curves:

(119) $$L_{\infty }=\frac{2\sigma_{\infty }}{p_{\alpha }-p_{sp}}\;,\;\mu_{\alpha }\left(T_{sp},p_{\alpha }\right)=\mu_{\beta }\left(T_{sp},p_{sp}\right)\;.$$

For any set of values $\left(T_{sp},p_{sp}\right)$, Equation (119) allows us to determine the appropriate value of the pressure in the critical cluster, $p_{\alpha }$, and as a consequence, the value of $L_{\infty }$. In its determination, we will follow here an approximative approach involving a Taylor expansion of the chemical potentials.

Going over to the determination of the parameter $L_{\infty }$ for the different cases analyzed, let us consider first, again, condensation and boiling at $T=T_{b}$ caused by variations of pressure, i.e., via a process characterized by the horizontal line in Figure 2 starting at the binodal and approaching the spinodal from one of both sides (see, again, also Figure 5 for an illustration). For such cases, we arrive at:

(120) $$L_{\infty }^{\left(p\right)}=\frac{2\sigma_{\infty }\left(T_{b},p_{b}\right)}{p_{\alpha }-p_{sp}}\;,\;\mu_{\alpha }\left(T_{b},p_{\alpha }\right)=\mu_{\beta }\left(T_{b},p_{sp}\right)\;,\;T=T_{b}\;.$$

Here, the superscript *p* in $L_{\infty }^{\left(p\right)}$ specifies again that the metastable state is created by the variation of pressure. By a Taylor expansion of the chemical potentials with respect to pressure in the vicinity of $p_{b}$, we get (with $\mu_{\alpha }\left(T_{b},p_{b}\right)=\mu_{\beta }\left(T_{b},p_{b}\right)$) approximately:

(121) $$p_{\alpha }-p_{sp}≅\left(p_{b}-p_{sp}\right)\left(1-\frac{{\tilde{v}}_{\beta }}{{\tilde{v}}_{\alpha }}\right)$$

and:

(122) $$L_{\infty }^{\left(p\right)}≅\frac{2\sigma_{\infty }\left(T_{b},p_{b}\right)}{\left(p_{b}-p_{sp}\right)}\frac{{\tilde{v}}_{\alpha }\left(T_{b},p_{b}\right)}{{\tilde{v}}_{\alpha }\left(T_{b},p_{b}\right)-{\tilde{v}}_{\beta }\left(T_{b},p_{b}\right)}\;,\;T=T_{b}\;.$$

For condensation and boiling at constant external pressure $p=p_{\beta }=p_{b}$ caused by the variation of temperature, we get instead (see, again, Figure 5 for an illustration):

(123) $$L_{\infty }^{\left(T\right)}=\frac{2\sigma_{\infty }\left(T_{b},p_{b}\right)}{p_{\alpha }-p_{b}}\;,\;\mu_{\alpha }\left(T_{sp},p_{\alpha }\right)=\mu_{\beta }\left(T_{sp},p_{b}\right)\;,\;p_{\beta }=p_{b}\;.$$

Here, the superscript *T* in $L_{\infty }^{\left(T\right)}$ specifies again that the metastable state is created by the variation of temperature. Employing the same method as in the derivation of Equation (122), we obtain instead:

(124) $$p_{\alpha }-p_{b}≅\left(T_{sp}-T_{b}\right)\left(\frac{{\tilde{s}}_{\alpha }-{\tilde{s}}_{\beta }}{{\tilde{v}}_{\alpha }}\right)$$

and:

(125) $$L_{\infty }^{\left(T\right)}≅\frac{2\sigma_{\infty }\left(T_{b},p_{b}\right)}{\left(T_{sp}-T_{b}\right)}\left(\frac{{\tilde{v}}_{\alpha }\left(T_{b},p_{b}\right)}{{\tilde{s}}_{\alpha }\left(T_{b},p_{b}\right)-{\tilde{s}}_{\beta }\left(T_{b},p_{b}\right)}\right)\;,\;p_{\beta }=p_{b}\;.$$

**Figure 7:**
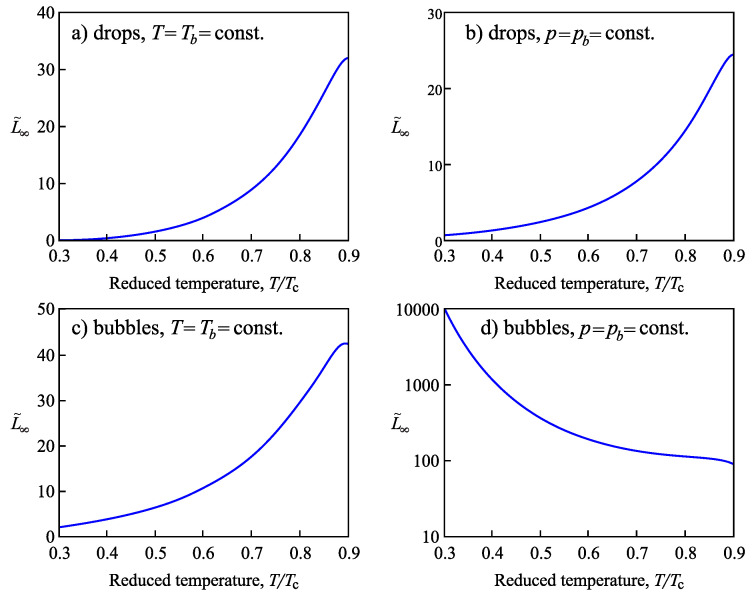
Correction parameters, $L_{\infty }$, in the expression for the curvature dependence of the surface tension, of critical droplets and bubbles, Equation (116), as defined by Equations (126) and (127). As the reference state, the temperature $T_{b}^{\mathrm{ref}}=0.7T_{c}$ and the corresponding value of pressure, $p_{b}^{\mathrm{ref}}$, along the binodal curve were chosen. The value of the surface tension at this reference state is taken equal to $\sigma_{\mathrm{ref}}=0.1\;J/m^{2}$, again. The correction parameter is expressed in dimensionless units, ${\tilde{L}}_{\infty }=\left|L_{\infty }\left(T_{b},p_{b}\right)\right|/\sqrt[{3}]{{\tilde{v}}_{l}\left(T_{b}^{\mathrm{ref}},p_{b}^{\mathrm{ref}}\right)}$, and since it enters Equation (103) as $L_{\infty }^{2}$, its absolute value is shown. The four different situations are illustrated at Figure 5. Note that the values of $T_{sp}$ and $p_{sp}$, entering Equations (126) and (127), are different for the different modes of the generation of metastable states.

In analogy to Equations (109) and (111), we obtain:

(126) $$L_{\infty }^{\left(p\right)}≅\frac{2\sigma_{\infty }\left(T_{b},p_{b}\right)}{\left(p_{b}-p_{sp}\right)}\left(\frac{{\tilde{v}}_{l}\left(T_{b},p_{b}\right)}{{\tilde{v}}_{l}\left(T_{b},p_{b}\right)-{\tilde{v}}_{v}\left(T_{b},p_{b}\right)}\right)\;\;,$$

$$L_{\infty }^{\left(T\right)}≅\frac{2\sigma_{\infty }\left(T_{b},p_{b}\right)}{\left(T_{sp}-T_{b}\right)}\left(\frac{{\tilde{v}}_{l}\left(T_{b},p_{b}\right)}{{\tilde{s}}_{l}\left(T_{b},p_{b}\right)-{\tilde{s}}_{v}\left(T_{b},p_{b}\right)}\right)$$

for droplets and:

(127) $$L_{\infty }^{\left(p\right)}≅\frac{2\sigma_{\infty }\left(T_{b},p_{b}\right)}{\left(p_{b}-p_{sp}\right)}\left(\frac{{\tilde{v}}_{v}\left(T_{b},p_{b}\right)}{{\tilde{v}}_{v}\left(T_{b},p_{b}\right)-{\tilde{v}}_{l}\left(T_{b},p_{b}\right)}\right)\;\;,$$

$$L_{\infty }^{\left(T\right)}≅\frac{2\sigma_{\infty }\left(T_{b},p_{b}\right)}{\left(T_{sp}-T_{b}\right)}\left(\frac{{\tilde{v}}_{v}\left(T_{b},p_{b}\right)}{{\tilde{s}}_{v}\left(T_{b},p_{b}\right)-{\tilde{s}}_{l}\left(T_{b},p_{b}\right)}\right)$$

for bubbles. The dependence of these parameters on temperature involving Equations (120)–(127) is illustrated in Figure 7.

**Figure 8:**
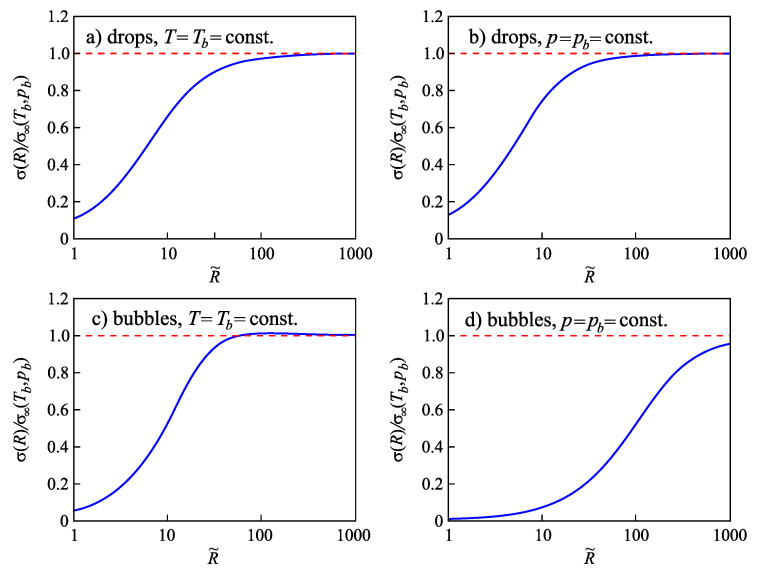
$\sigma \left(R\right)/\sigma_{\infty }\left(T_{b},p_{b}\right)$ for four different cases of phase formation. Here, we consider one reference state $T=0.7T_{c}$ and the respective value of pressure, $p_{b}$, and compute the dependence σ(R) for boiling and condensation both if pressure or temperature is changed. The reference value of the surface tension is taken equal to $\sigma^{\mathrm{ref}}=\sigma_{\infty }\left(T_{b}=0.7T_{c},p_{b}=p_{b}\left(0.7T_{c}\right)\right)=0.1$ J/m${}^{2}$, again. The radius is shown in dimensionless units $\tilde{R}=R/d$ with $d=\sqrt[{3}]{{\tilde{v}}_{l}\left(T_{b}^{\mathrm{ref}},p_{b}^{\mathrm{ref}}\right)}=3.515\cdot 10^{-10}$ m.

Once we have determined $\delta_{\infty }$ and $L_{\infty }$, we can arrive at the dependencies for σ(R) on *R* as described via Equation (116) for the possible different paths of the generation of metastable states. As an example, we consider condensation and boiling for one reference state $T=0.7T_{c}$ and the respective value of pressure and consider the dependence σ=σ(R) for boiling and condensation both if pressure and temperature are changed. The results are illustrated in Figure 8. In contrast to crystallization, here, the correction term in the expression for the curvature dependence of the surface tension is essential to arrive at a correct description in the whole range of metastable states. In the application to the experiment, the method has, similarly to crystallization, the huge advantage that for the determination of the parameters $\delta_{\infty }$ and $L_{\infty }$, only directly-measurable parameters of both fluid phases are required. This feature results in huge advantages in the application to the interpretation of experimental data, in particular, on nucleation.

Note that the same method can be employed always if a spinodal exists for the system under consideration, like, e.g., in segregation processes in solutions [106,107] or crystallization in multi-component systems provided the composition of the newly-formed crystalline aggregates depend on pressure and/or temperature, and/or the size of the crystal clusters (as noted, if, at least, one of these conditions is not fulfilled, then there does not exist a spinodal in melt crystallization [7,25,26]).

### 4.3. Comparison with Density Functional Studies: The van der Waals Approach

#### 4.3.1. Some Introductory Comments

In the preceding sections, we employed the classical Gibbs’ theory of surface phenomena and their consequences in order to arrive at a new general relation for the curvature dependence of the surface tension of drops and bubbles covering metastable initial states in the whole range from the binodal to the spinodal curves. It involves a well-founded assumption, formulated already by Gibbs [1], that the work of critical cluster formation tends to zero at the spinodal curve.

Reinventing the van der Waals approach [44,45] in the description of inhomogeneous systems, Cahn and Hilliard [108] reconfirmed this result and developed an alternative method of description of the properties of critical clusters and their properties. These results were somewhat later advanced by Lifshitz and Kagan [109]. The van der Waals approach and more advanced density functional computations lead to different predictions concerning the properties of critical clusters and their sizes in dependence on supersaturation as compared with Gibbs’ classical method. These differences could be reconciled by us advancing a generalization of Gibbs’ classical treatment first in application to segregation processes in solutions [106,107] and then to the description of condensation and boiling [103,104,105]. In agreement with density functional approaches, the work of critical cluster formation is shown to approach zero at the spinodal, but the size of the critical clusters tends to infinity. Furthermore, the bulk state parameters of the critical clusters are different when compared with the predictions of Gibbs’ classical approach: the bulk state parameters of the critical clusters approach the respective values of the ambient phase. As a consequence, both thermodynamic driving force and surface tension tend to zero. A detailed discussion is given in the cited papers.

In the present paper, we will concentrate on only one topic. We analyze the question whether the work of critical cluster formation obtained via density functional computations can be described appropriately in terms of Gibbs’ classical theory utilizing the new equation advanced by us for the curvature dependence of the surface tension of critical droplets and bubbles, Equation (116). In more detail, the procedure is the same as described in Section 3.1. We compute the dependence of the work of critical cluster formation on the degree of supersaturation and check whether it can be interpreted, determining the bulk properties of the critical clusters via Gibbs’ theory and the surface tension via Equation (116).

#### 4.3.2. Planar Interfaces

The Gibbs or Helmholtz free energies of an inhomogeneous system with a planar interface can be written in terms of van der Waals’ theory of capillarity as a functional [44,45]:

(128) $$F=A\int_{-\infty }^{+\infty }\left[f\left(\rho \right)+\kappa {\left(\frac{d\rho }{dz}\right)}^{2}\right]\;dz\;.$$

Here, we employ a system of coordinates where the (x,y)-plane is located in the interfacial zone and the *z*-axis is located perpendicular to the interface; with an increase of *z*, we go over from the liquid to the vapor. In Equation (128), *A* is the surface area, f(ρ) the volume density of Gibbs’ free energy, and κ a coefficient determining the magnitude of interfacial effects. For the bulk density of the Gibbs’ free energy, we have:

(129) $$f\left(\rho \right)=-\frac{3}{2}\rho \;k_{B}T\ln\left(\frac{2\pi \;m\;k_{B}T}{h^{2}}\right)-\rho \;k_{B}T\ln\left(\frac{1-b\;\rho }{\rho }\right)-\rho \;k_{B}T-a\;\rho^{2}\;,$$

where the parameters *a* and *b* are determined by the interaction potential of the particles. In a system of hard spheres with the Katz potential:

(130) $$\varphi \left(\tilde{r}\right)=\left\{\begin{array}{cc} \infty \;, & \tilde{r}\leq d\\ -\varepsilon \exp\left(-\frac{\tilde{r}}{d}\right)\;, & \tilde{r}>d \end{array}\right.\;,$$

we obtain:

(131) $$b=22/33\pi \;d^{3},$$

(132) $$a=-2\pi \int \varphi \left(\tilde{r}\right){\tilde{r}}^{2}d\;\tilde{r}=15b\;\varepsilon \;e\;.$$

Here, *d* is a parameter determining the size of the hard spheres. In the mean-field approach utilized in the derivation of Equation (129), the coefficient κ does not depend on thermodynamic parameters and can be computed via the interaction potential, resulting in:

(133) $$\kappa =-\frac{\pi }{3}\int {\varphi \left(\tilde{r}\right){\tilde{r}}^{4}d\;\tilde{r}=\frac{13}{6}a\;\left(\frac{3b}{2\pi }\right)}^{2/3}\;.$$

For the surface tension at a planar interface, we may write generally:

(134) σ∞=F−F′−F″F−F′−F″AA=f−f′−f″=∫−∞+∞Δf+κdρdz2dz,

(135) $$\Delta \;f=f-f^{\prime \prime }-\left(\rho -\rho^{\prime \prime }\right){\left.\frac{\partial \;f}{\partial \rho }\right|}_{\rho =\rho^{\prime \prime }}\;.$$

Here, $F^{\prime }$ and $F^{\prime \prime }$ are the Gibbs free energies of the liquid and gas phases in the condition that the respective phases remain homogeneous up to the dividing surface. By minimization of the functional, Equation (134), we obtain the density profile in the interfacial region:

(136) z=∫ρ″ρκκΔfΔf1/2dρ

and the surface tension in the form:

(137) $$\sigma_{\infty }=2\int_{-\infty }^{+\infty }\Delta \;f\;dz=2\int_{\rho^{\prime \prime }}^{\rho^{\prime }}{\left(\kappa \;\Delta \;f\right)}^{1/2}\;d\rho \;.$$

Similarly to Equation (77), we will now utilize reduced thermodynamic parameters:

(138) $$\Pi \equiv \frac{p}{p_{c}}\;,\;\theta \equiv \frac{T}{T_{c}}\;,\;\psi \equiv \frac{\rho }{\rho_{c}}\;,$$

(139) ϕ=ffc,γ=σρc2/3kBTc,ξ=zρc1/3,λ=κ3kBTc3kBTc8ρc5/38ρc5/3.

The critical values of the respective quantities are defined as:

(140) $$T_{c}=\frac{8a}{27k_{B}b}\;,\;p_{c}=f_{c}=\frac{a}{27b^{2}}\;,\;\rho_{c}=\frac{1}{3b}\;.$$

In such reduced parameters, we obtain the following expression for the surface tension:

(141) $$\gamma =\left(\frac{3}{4}\right)\lambda^{1/2}\int_{\psi^{\prime \prime }}^{\psi^{\prime }}{\left(\Delta \;\phi \right)}^{1/2}\;d\psi \;,$$

where:

(142) $$\Delta \;\phi =\frac{8}{3}\psi \theta \ln\frac{\psi \left(3-\psi^{\prime \prime }\right)}{\psi^{\prime \prime }\left(3-\psi \right)}+8\theta \frac{\psi^{\prime \prime }-\psi }{3-\psi^{\prime \prime }}-3{\left(\psi^{\prime \prime }-\psi \right)}^{2}\;.$$

**Figure 9:**
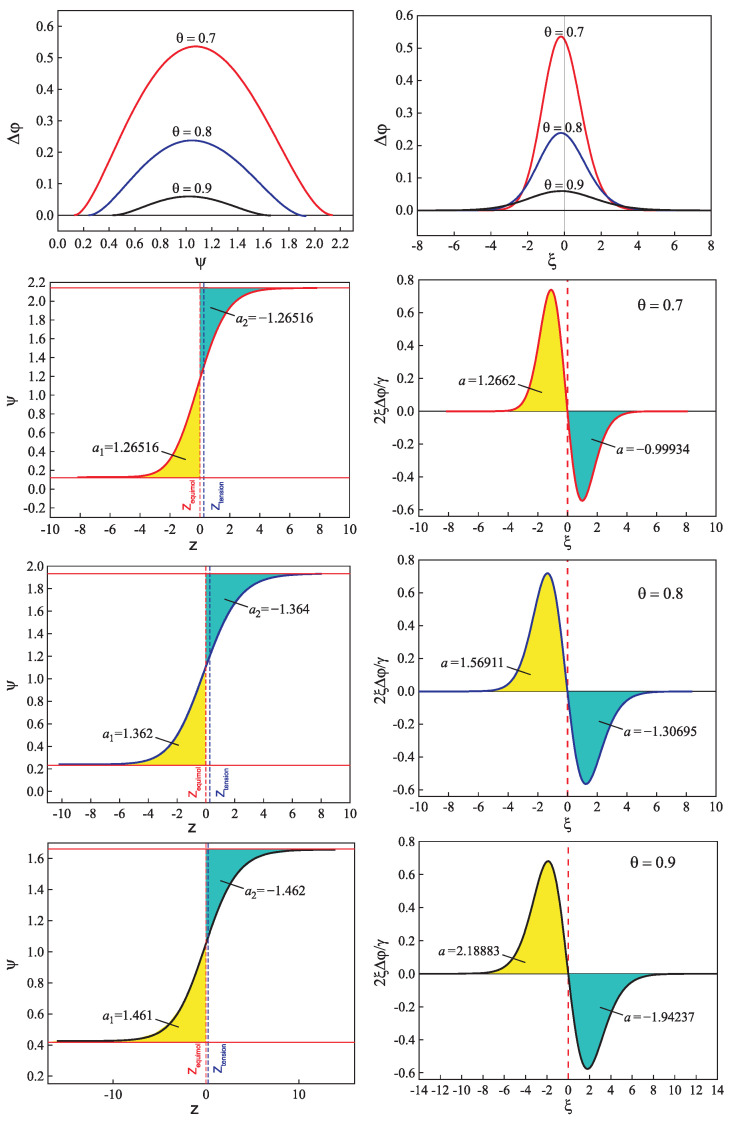
Results of the computations (see the text). By *a*, $a_{1}$, and $a_{2}$, the surface area is denoted as indicated in the figure.

**Figure 10:**
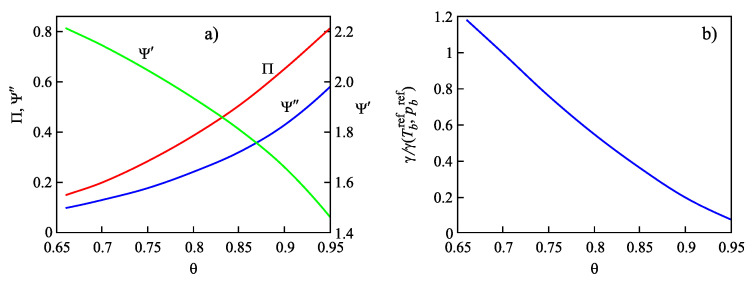
Illustration of the results given in Table 2. The dependence of the surface tension on temperature for states along the binodal is shown in Figure 9b in a form similar to the results shown in Figure 4. Again, T=0.7 is taken as the reference state, and the surface tension is shown as $\gamma \left(T_{b},p_{b}\right)/\gamma \left(T_{b}^{\mathrm{ref}},p_{b}^{\mathrm{ref}}\right)$ versus temperature using the results given in the table.

The results of the computations of the pressure, the densities, and the surface tension are given in Table 2 and are illustrated in Figure 9 and Figure 10.

#### 4.3.3. Determination of the Tolman Parameter in van der Waals’ Theory of Capillarity

According to its definition, Equation (2), the value of the Tolman parameter for a planar interface in the chosen system of coordinates is given by:

(143) $$\delta_{\infty }=z_{e}-z_{t}\;.$$

Here, $z_{e}$ describes the location of the equimolecular dividing surface and $z_{t}$ the location of the surface tension. Utilizing the notations outlined in Section 4.3.2, Equation (136) may be written as:

(144) $$\xi =\lambda^{1/2}\int_{\psi^{\prime \prime }}^{\psi^{\prime }}{\left(\Delta \;\phi \right)}^{-1/2}\;d\psi .$$

For the Katz potential, the parameter κ is given by Equation (133) and can be written in a reduced form as:

(145) $$\lambda =\frac{13}{2{\left(2\pi \right)}^{2/3}}\;.$$

Utilizing Equation (144), we determine the density profile in the planar interfacial zone at temperatures θ=0.7, 0.8, and 0.9. For such a purpose, we separate the integral into two terms as:

(146) $$I=\int_{\psi^{\prime \prime }}^{\psi^{\prime }}{\left(\Delta \;\phi \right)}^{-1/2}\;d\psi +\int_{\psi_{0}}^{\psi^{\prime }}{\left(\Delta \;\phi \right)}^{-1/2}\;d\psi =I_{1}+I_{2}\;,$$

where as a first approximation, we may write ψ0=ψ′+ψ″ψ′+ψ″22. By varying the value of $\psi_{0}$, we may reach the equality of the values of both integrals, $I_{1}=I_{2}$. The value of $\psi_{0}$, at which this equation holds, determines the location of equimolecular dividing surface. We now move the origin of the system of coordinates into the equimolecular dividing surface. With such a choice of the reference system, we get $z_{e}=0$. The location of the surface tension, $z_{t}$, is given then by:

(147) $$\xi {\;}_{t}=\frac{2}{\gamma_{\infty }}\int_{-\infty }^{+\infty }\xi \;\Delta \;\phi \;\;d\xi$$

and the value of the Tolman parameter is obtained as:

(148) $$\delta_{\infty }\rho_{c}^{1/3}=-\xi {\;}_{t}\;.$$

The results of the computations are given in Table 3. Utilizing Equation (3) for the description of the surface tension, it results in a negative value of the Tolman parameter for droplets and a positive value of this parameter for bubbles, both of critical sizes.

#### 4.3.4. Dependence of the Surface Tension of Bubbles and Droplets on the Radius of the Dividing Surface

As already discussed earlier (Equations (6) and (44)), the equilibrium conditions can be written in the framework of Gibbs’ theory as:

(149) $$\mu_{\alpha }\left(p_{\alpha },T\right)=\mu_{\beta }\left(p_{\beta },T\right)\;,\;p_{\alpha }-p_{\beta }=\frac{2\sigma }{R}\;.$$

Here, the subscript α specifies the parameters of the newly-evolving phase and β the parameters of the ambient phase where the clusters of the newly-evolving phase are formed. In the framework of van der Waals’ theory of capillarity, the condition of the equilibrium of a bubble or a droplet is given by [44,45]:

(150) $$2\kappa \left(\frac{d^{2}\rho }{dr^{2}}\right)+\frac{4\kappa }{r}\left(\frac{d\rho }{dr}\right)=\mu \left(\rho \right)-\mu_{\beta }\;,$$

where the boundary conditions:

(151) $$\begin{array}{ccc} \rho \to \rho_{\beta } & \;\mathrm{at}\; & r\to \infty \\ \frac{d\rho }{dr}\to 0 & \;\mathrm{at}\; & r\to \infty \end{array}$$

have to be fulfilled. Here, ρ(r) is the local density and $\rho_{\beta }$ the density of the ambient phase. The coefficient κ we consider as independent of density, ρ, and *r*.

The work of critical cluster formation is given in this case by:

(152) $$W=\min\max\Delta F\left\{\rho \right\}\;,$$

where ΔF is the change of the Helmholtz free energy caused by the formation of the critical bubble or droplet:

(153) $$\Delta F\left\{\rho \right\}=\int_{V}\left[\Delta f+\kappa {\left(\nabla \rho \right)}^{2}\right]dV\;,$$

(154) $$\Delta \;f=f-f_{\beta }-\left(\rho -\rho_{\beta }\right){\left.\frac{\partial f}{\partial \rho }\right|}_{\;\rho =\rho_{\beta }}\;.$$

In contrast to Gibbs’ approach (cf. Equation (7)), in the determination of the work of critical cluster formation, no assumption concerning the homogeneity of the cluster phase is made here. Employing Equations (152)–(154) and Gibbs’ result, Equation (7), we can compute the value of the surface tension via the relation:

(155) $$\sigma ={\left(\frac{3}{16\pi }\right)}^{1/3}{\left(p_{\alpha }-p_{\beta }\right)}^{2/3}W^{1/3}\;.$$

Here, for any value of the pressure in the ambient phase, the pressure in the critical cluster is determined via Equation (149). Its radius is given by:

(156) $$R=\frac{2\sigma }{p_{\alpha }-p_{\beta }}\;.$$

Using the reduced parameters θ, Π, ψ, γ, and λ introduced with Equations (138) and (139) and, in addition,

(157) x=rρc1/3,F=ffpcpc,w=WW3kBTc3kBTc883kBTc3kBTc88,

the basic equation employed in the computations can be written as:

(158) $$\gamma ={\left(\frac{3}{16\pi }\right)}^{1/3}\frac{3}{8}w^{1/3}{\left(\Pi_{\alpha }-\Pi_{\beta }\right)}^{2/3}\;,$$

(159) $$x=\frac{16\gamma }{3\left(\Pi_{\alpha }-\Pi_{\beta }\right)}\;,\;\lambda =\frac{13}{2}{\left(2\pi \right)}^{-2/3}\;,$$

(160) $$\frac{d^{2}\psi }{dx^{2}}+\frac{2}{\chi }\frac{d\psi }{d\chi }=\frac{1}{2\lambda }\frac{\partial \Delta F}{\partial \psi }\;,$$

(161) $$\frac{\partial \Delta F}{\partial \psi }=8\theta \left(\frac{1}{3-\psi }-\frac{1}{3-\psi_{\beta }}\right)+6\left(\psi -\psi_{\beta }\right)+\frac{8}{3}\theta \ln\left[\frac{3-\psi_{\beta }}{\psi_{\beta }}\cdot \frac{\psi }{3-\psi }\right].$$

By a Taylor expansion of the right-hand side of Equation (160) in the vicinity of the density of the ambient phase, $\psi_{\beta }$, we may obtain a solution of this relation valid for sufficiently large distances from the center of the critical cluster as:

(162) $$\psi -\psi_{\beta }≅\frac{a_{1}}{x}\exp\left(-a_{2}x\right)\;.$$

In the general case, Equation (160) can be solved numerically only. For this purpose, we used the Runge–Kutta method. The integration was performed with a step δx=0.05 starting from the center of the critical cluster. The initial distribution $\psi_{0}$ was selected arbitrarily, and the derivative ${\left.d\psi /dx\right|}_{x=0}$ was taken equal to zero, in line with Equation (151). At large values of *x*, when the density in the critical cluster should approach $\psi_{\beta }$, the solution of Equation (160) was demanded to be described by Equation (162). If this condition was not fulfilled, another initial distribution was selected. The procedure of the selection of appropriate initial distributions was terminated if the solutions did not differ from each other by less than 0.01% of the initial density.

**Figure 11:**
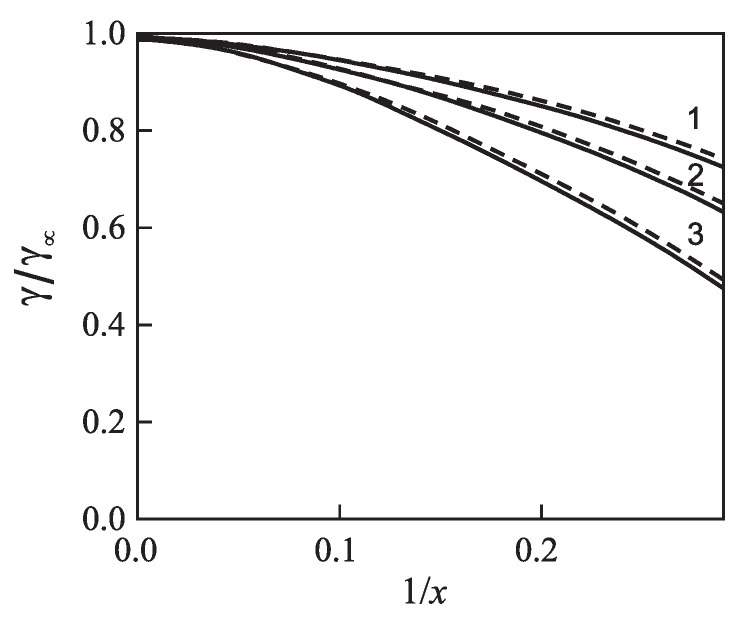
Dependence of the surface tension of critical bubbles on the inverse of the radius (in reduced units) in a superheated liquid at temperatures (1) θ=0.7, (2) θ=0.8, and (3) θ=0.9. Full curves are the results of numerical computations via the van der Waals method, and dashed curves are approximations as described via Equation (163).

**Figure 12:**
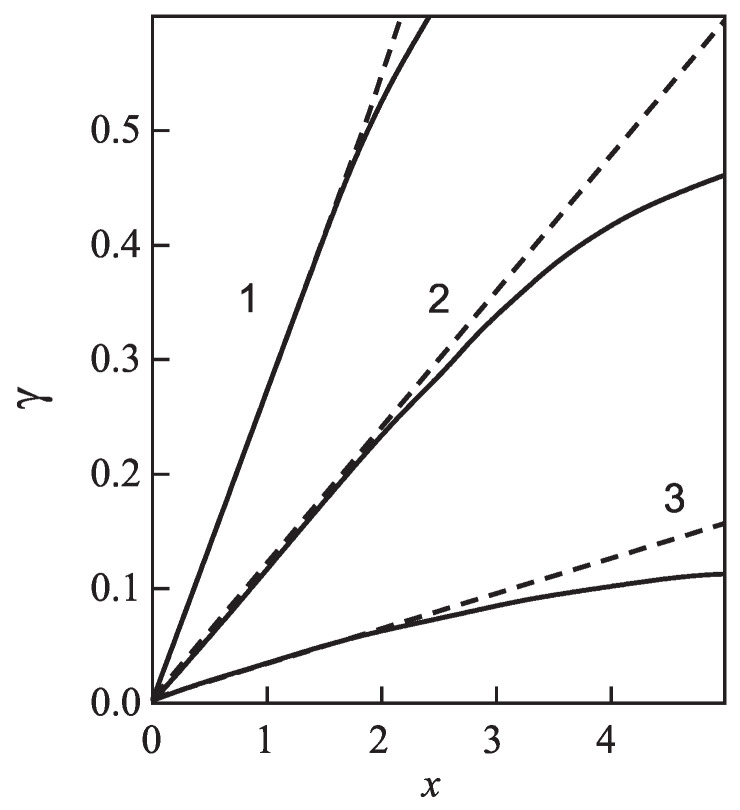
Dependence of the surface tension of critical bubbles on the radius (in reduced units) in a superheated liquid at temperatures (1) θ=0.7, (2) θ=0.8, and (3) θ=0.9 near the spinodal curve. Full curves are the results of numerical computations via the van der Waals method, and dashed curves are approximations as described via Equation (114).

Results of the computations are given on Figure 11 and Figure 12. In Figure 11, the ratio $\gamma /\gamma_{\infty }$ is shown with dependence on the inverse of the radius for the surface tension for large radii of the critical bubbles. The full curves are the results of the numerical computations, and the dashed curve is obtained from equation:

(163) $$\gamma =\frac{\gamma_{\infty }}{1+\frac{\delta_{T}}{x}+\frac{l}{x^{2}}}\;.$$

In Figure 12, similar results are shown for the state near the spinodal curve, being in line with the predictions of Equation (114).

**Figure 13:**
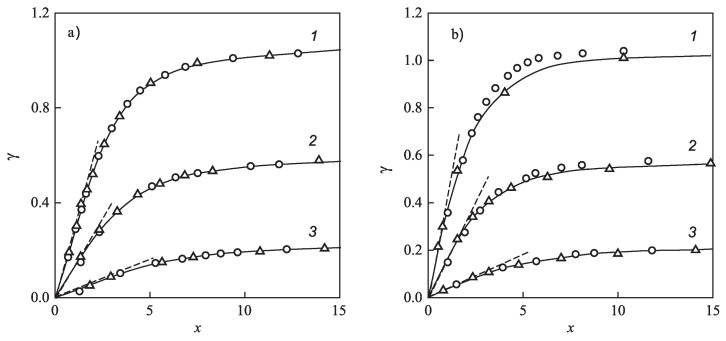
Dependence of the surface tension, γ, of (**a**) bubbles and (**b**) droplets on curvature for three different values of temperature, (1) θ=0.7, (2) θ=0.8, and (3) θ=0.9. Triangles and circles show results of computations by van der Waals square density functional computations. The full curves, based on Equation (116), result in an excellent fit of the data. By dashed curves, the limiting behavior of this dependence is shown to be in line with Equation (118) and the results presented in Figure 12.

In Figure 13, the dependence of the surface tension, γ, of (a) bubbles and (b) droplets on curvature is shown for three different values of temperature. Triangles and circles show the results of computations by van der Waals square density functional computations. The full curves, based on Equation (116), result in an excellent fit of the data. By dashed curves, the limiting behavior of this dependence is shown to be in line with Equation (118) and the results presented in Figure 12. In the specification of the parameters in Equation (116), the Tolman parameter, $\delta_{\infty }$, was taken from Table 3 determined via van der Waals square gradient computations. The parameter *l* was chosen as a fit parameter of the results obtained for the surface tension via the van der Waals method. The fit was performed for bubbles at values x=2.3, x=3.23, x=3.38 and for droplets at x=2.28, x=3.18, x=3.12 for the reduced temperatures, (1) θ=0.7, (2) θ=0.8, and (3) θ=0.9, correspondingly.

Comparing the results obtained via application of the Stefan–Skapski–Turnbull relation and the van der Waals approach, we can conclude: (i) The theoretical predictions for the dependence of the surface tension on temperature and pressure along the binodal curve are qualitatively and even quantitatively widely identical (Figure 4 and Figure 10). (ii) The predictions for the Tolman parameter yield values with similar orders of magnitude. The sign is, however, different, at least, for some of the cases considered. Such differences of the sign of the Tolman parameter obtained by different methods of computation are known and widely discussed in the literature (e.g., [8,9,10,11,12,13,16,17,18,22,27,28]). We will not repeat them here, but draw attention to another consequence of the studies performed by us: (iii) The dependencies of the surface tension on cluster size in the whole range of metastable states are widely similar for droplets and bubbles and show the same behavior for both methods of computations, as described here (see Figure 8 and Figure 13). They are well-described by Equation (116) proposed in the present paper. For applications to nucleation theory, this is the major consequence, and in this respect, both methods are widely equivalent in their results. Anyway, nucleation proceeds at sufficiently large supersaturation where the particular value of the Tolman parameter is already not the dominating factor for the dependence of the surface tension on curvature in the application to condensation and boiling. The method based on the Stefan–Skapski–Turnbull relation has, however, the major advantage already mentioned that in the determination of the dependence σ=σ(R), only directly-measurable properties of the fluids under consideration are required. This feature considerably simplifies its application in the interpretation of experimental data.

## 5. Results and Discussion

The main results of the present analysis can be summarized in the following way: (i) Based on the approach utilized in [2], in previous papers [7,25,26,48], expressions for the thermodynamic driving force of crystal nucleation in multi-component systems in dependence on pressure and temperature were derived involving only directly-measurable thermodynamic parameters of both phases under consideration (Equation (10)). In the analysis, two approximations were made: the composition and/or structure of the newly-evolving crystal phase is assumed to be independent of pressure and temperature, and the volume of the crystal phase is supposed to be nearly equal to the volume of the liquid undergoing crystallization and to depend only weakly on composition. The latter assumption was made in all cases, except when differences of these quantities were considered. The first assumption is an essential ingredient of CNT. The second assumption gives the possibility to arrive at such general relations valid for the description of both stoichiometric and non-stoichiometric crystallization. The second assumption can be omitted for the analysis of crystallization in one-component systems. Employing the same approach as in the cited papers, we formulated here these more precise relations for the thermodynamic driving force in one-component systems (Equations (15) and (27)). (ii) Relying on the Stefan–Skapski–Turnbull relation and utilizing the same method as in the determination of the thermodynamic driving force, expressions for the dependence of the surface tension on pressure and temperature for crystal nucleation in multi-component systems, Equation (44), were derived in [24,25,26]. This method was advanced here to a more precise formulation of the pressure and temperature dependence of the surface tension for phase formation in one-component systems, Equation (35), proceeding in a similar way as in the analysis of the thermodynamic driving force of phase formation. (iii) It was shown that the size or curvature dependence of the surface tension can be described by the Gibbs–Tolman relations, Equations (1) and (3), when either pressure or temperature was changed and the composition and/or structure of the ambient and crystalline phases does not depend on the variations of these external control parameters. The Tolman parameter has different values in dependence on whether either temperature (Equations (51) and (58)) or pressure (Equations (47) and (56)) was varied. If the mentioned conditions are not fulfilled, the Tolman approach cannot be employed, in general, for the description of the size dependence of the surface tension of critical clusters (e.g., Equations (53), (59)–(61)). (iv) Critical crystal clusters are, in general, not spheres, but have a shape determined by the Gibbs–Curie–Wulff theorem. Despite that, as demonstrated here in two different ways, they can be described in terms of Gibbs’ theory by a model assuming a spherical cluster with a radius, *R*, and a (average) value of the surface tension, σ. This is the basis for applying the Gibbs–Tolman approach to the description of the size or curvature dependence of the surface tension for crystallites of critical sizes. (v) Utilizing such a model approach and the relations for the dependence of thermodynamic driving force and surface tension on pressure and temperature, expressions for the Tolman parameter for crystallization in multi-component systems were formulated for the cases when either temperature (Equation (69)) or pressure (Equation (70)) are varied. The results were shown to be in good agreement with experimental data on crystallization in both one- and multi-component systems. Consequently, the Stefan–Skapski–Turnbull relation allows one to arrive at an estimate for the Tolman parameter for the description of crystallization in multi-component liquids (Table 1). It resulted in the possibility to interpret experimental data for crystallization in real multi-component systems employing only one fit parameter (the value of the surface tension for a planar equilibrium coexistence of liquid and crystal), but accounting for a size or curvature dependence of the surface tension. As noted, the account of such dependence is a necessary requirement for an appropriate description of crystal nucleation [6,15,32,57,72]. (vi) The Stefan–Skapski–Turnbull relation was further employed here in the description of the surface tension of droplets and bubbles in condensation and boiling (Equation (96)). It allowed us to determine the value of the surface tension along the binodal curve, provided its value for one of the states along this curve is known (Figure 4). (vii) In application to the description of the properties of critical clusters, the Tolman parameters were determined for both cases of phase formation caused by either variation of pressure or temperature (Equations (109) and (110), respectively, Equations (111) and (113)). (viii) Based on a suggestion formulated by one of us earlier [12,13], a generalization of the Tolman equation was proposed (Equation (116)) allowing one to describe the curvature dependence of the surface tension in the whole range of metastable state from the binodal to the spinodal curves. It yielded the theoretically-expected asymptotic behavior near the binodal and the spinodal curves (Equations (114), (117) and (118)). (ix) Analytical estimates were formulated also for the second parameter, $L_{\infty }$, entering Equation (116), and its value was determined for different cases of condensation and boiling. Having at one’s disposal the values of both parameters, $\delta_{\infty }$ and $L_{\infty }$, a new tool was developed for the interpretation of nucleation processes in condensation and boiling. (x) The results for the description of condensation and boiling were compared with square gradient density functional computations. In particular, we would like to note that the application of the Stefan–Skapski–Turnbull relation led to widely-equivalent results for the dependence of the surface tension on temperature along the binodal curve (Figure 4 and Figure 9) and for the dependencies σ=σ(R) in the whole range of metastable states (Figure 8 and Figure 13). The Stefan–Skapski–Turnbull approach may be employed consequently, at least, as a first estimate for such a dependence, not only for crystallization, but also for condensation and boiling. (xi) The method for the determination of the curvature dependence of the surface tension for condensation and boiling is closely connected with the existence of a spinodal curve. Consequently, it can be employed similarly to segregation processes in solutions and in processes of crystallization when, beyond the basic assumptions employed in CNT, the properties of the critical clusters depend significantly on pressure and temperature. The implementation of our approach to the analysis of this circle of problems will be discussed in detail in a forthcoming contribution.

## Figures and Tables

**Table 1:** Values of the parameters determining the crystal nucleation rates obtained in different ways as described in the text and in more detail in [15]. $\sigma_{\infty }$ and δ given in the second and fourth columns of the table were obtained in [32,33,34] as fit parameters in order to achieve the best fit of experimental data on steady-state nucleation rates to theoretical predictions utilizing Equation (3) for the description of the curvature dependence of the surface tension. In the approach employed here, $\delta_{\infty }$ is determined via Equation (69). The respective data are given in the sixth column of the table. The parameters are computed for $22.4{\mathrm{Na}}_{2}O\cdot 28.0\mathrm{CaO}\cdot 49.6{\mathrm{SiO}}_{2}$ (1N1C2S) [93], ${\mathrm{Na}}_{2}O\cdot 2\mathrm{CaO}\cdot 3{\mathrm{SiO}}_{2}$ (1N2C3S) [94,95,96], $2{\mathrm{Na}}_{2}O\cdot 1\mathrm{CaO}\cdot 3{\mathrm{SiO}}_{2}$ (2N1C3S) [97], ${\mathrm{Li}}_{2}O\cdot 2{\mathrm{SiO}}_{2}$ (L2S) [98], and $\mathrm{BaO}\cdot 2{\mathrm{SiO}}_{2}$ (B2S) [99]. The data required for the calculations are taken from cited papers.

Glass	Fit of Nucleation Rate Data				Equation (69)
	$\sigma_{\infty }\;\left[\frac{J}{m^{2}}\right]$	$d_{0}$ (nm)	$\frac{\delta }{d_{0}}$	$\left(\frac{d_{0}}{\delta }\right)\sigma_{\infty }\;\left[\frac{J}{m^{2}}\right]$	$\left(\frac{d_{0}}{\delta_{\infty }}\right)\sigma_{\infty }\;\left[\frac{J}{m^{2}}\right]$
1N1C2S	0.243	0.588	1.15	0.21	0.17
1N2C3S	0.235	0.588	1.1	0.214	0.17
2N1C3S	0.225	0.599	1.7	0.133	0.118
L2S	0.238	0.480	0.455	0.524	0.24
B2S	0.197	0.496	1.04	0.189	0.112

**Table 2:** Results of the computations of the pressure, Π, the densities, $\psi^{\prime }$ and $\psi^{\prime \prime }$, in the liquid and vapor phases, and of the surface tension, γ.

θ	Π	$\psi^{\prime }$	$\psi^{\prime \prime }$	γ
0.66	0.147	2.212	0.096	1.26
0.70	0.200	2.140	0.128	1.06
0.75	0.283	2.042	0.177	0.81
0.80	0.383	1.933	0.240	0.58
0.85	0.504	1.807	0.320	0.38
0.90	0.647	1.657	0.426	0.21
0.95	0.812	1.462	0.579	0.075

**Table 3:** Results of computations (see the text).

θ	$\psi^{\prime }$	$\psi^{\prime \prime }$	γ	$\delta_{\infty }$
0.7	2.14035	0.12802	1.06	−0.1335
0.8	1.93334	0.239667	0.58	−0.13108
0.9	1.657	0.426	0.21	−0.123235
